# Connexin 43 phosphorylation by casein kinase 1 is essential for the cardioprotection by ischemic preconditioning

**DOI:** 10.1007/s00395-021-00861-z

**Published:** 2021-03-22

**Authors:** Christine Hirschhäuser, Alessio Lissoni, Philipp Maximilian Görge, Paul D. Lampe, Jacqueline Heger, Klaus-Dieter Schlüter, Luc Leybaert, Rainer Schulz, Kerstin Boengler

**Affiliations:** 1 Institut für Physiologie, Justus-Liebig Universität Gießen, Aulweg 129, 35392 Giessen, Germany; 2 Department of Basic Medical Sciences, Physiology group, Faculty of Medicine and Health Sciences, Ghent University, Ghent, Belgium; 3 Fred Hutchinson Cancer Research Center, Seattle, WA USA

**Keywords:** Connexin, Phosphorylation, Ischemia/reperfusion, Ischemic preconditioning, Mitochondria, Hemichannel

## Abstract

Myocardial connexin 43 (Cx43) forms gap junctions and hemichannels, and is also present within subsarcolemmal mitochondria. The protein is phosphorylated by several kinases including mitogen-activated protein kinase (MAPK), protein kinase C (PKC), and casein kinase 1 (CK1). A reduction in Cx43 content abrogates myocardial infarct size reduction by ischemic preconditioning (IPC). The present study characterizes the contribution of Cx43 phosphorylation towards mitochondrial function, hemichannel activity, and the cardioprotection by IPC in wild-type (WT) mice and in mice in which Cx43-phosphorylation sites targeted by above kinases are mutated to non-phosphorylatable residues (Cx43^MAPKmut^, Cx43^PKCmut^, and Cx43^CK1mut^ mice). The amount of Cx43 in the left ventricle and in mitochondria was reduced in all mutant strains compared to WT mice and Cx43 phosphorylation was altered at residues not directly targeted by the mutations. Whereas complex 1 respiration was reduced in all strains, complex 2 respiration was decreased in Cx43^CK1mut^ mice only. In Cx43 epitope-mutated mice, formation of reactive oxygen species and opening of the mitochondrial permeability transition pore were not affected. The hemichannel open probability was reduced in Cx43^PKCmut^ and Cx43^CK1mut^ but not in Cx43^MAPKmut^ cardiomyocytes. Infarct size in isolated saline-perfused hearts after ischemia/reperfusion (45 min/120 min) was comparable between genotypes and was significantly reduced by IPC (3 × 3 min ischemia/5 min reperfusion) in WT, Cx43^MAPKmut^, and Cx43^PKCmut^, but not in Cx43^CK1mut^ mice, an effect independent from the amount of Cx43 and the probability of hemichannel opening. Taken together, our study shows that alterations of Cx43 phosphorylation affect specific cellular functions and highlights the importance of Cx43 phosphorylation by CK1 for IPC’s cardioprotection.

**Supplementary Information:**

The online version contains supplementary material available at 10.1007/s00395-021-00861-z.

## Introduction

Cell–cell communication in the ventricular myocardium is mediated via gap junctions, which are mainly formed by connexin 43 (Cx43). Six Cx43 proteins assemble into hexamers, the so-called connexons or hemichannels, and are transported to the sarcolemma, where two hemichannels from adjacent cells dock to each other and form gap junctions, which allow the passage of molecules up to 1.5 kDa in size and mediate electrical and metabolic cell coupling. Cx43 is part of a family comprising 21 members in humans with molecular weights ranging from 26 to 60 kDa and which are expressed in different organs and cell types. The connexin secondary structure consists of four transmembrane domains and a cytosolic amino-and carboxyterminus. Within the carboxyterminus of Cx43, at least 21 serine (S) residues are targeted and phosphorylated by different kinases, among them protein kinase B (PKB or AKT), protein kinase C (PKC), casein kinase 1 (CK1), calcium/calmodulin kinase II (CamKII), mitogen-activated protein kinase (MAPK), p34^cdc2^/cyclin B kinase (p34cdc2), and the pp60src kinase (src) (for review, see [69]).

S262 (similar to S279 and S282) is commonly suggested to be targeted by the extracellular-signal-related kinase (ERK) and the mutation of S262 to alanine increases gap junction permeability and inhibits desoxyribonucleic acid (DNA) synthesis. CK1 phosphorylates Cx43 at S325, S328, and S330 and mice in which S325/328/330 are mutated to alanines display disturbed gap junction formation and are susceptible to arrhythmias [54]. After phosphorylation of S373 by AKT, the interaction between Cx43 and zonula occludens 1 (ZO-1) is limited and the size of gap junctions is increased which may represent a first step in the disassembly process of gap junctions. Cx43 phosphorylation at S365 is also involved in the protein–protein interaction with ZO-1. Dephosphorylation of Cx43 at S365 is essential for the phosphorylation of Cx43 at the PKC-target site S368; therefore, the Cx43 phosphorylation at S365 is suggested to function as a “gatekeeper” [71]. Cx43 S365 phosphorylation is increased under conditions where the activity of protein kinase A (PKA) is enhanced; however, Cx43 is a poor substrate for a direct phosphorylation by PKA. PKC directly phosphorylates Cx43 at S368 and this phosphorylation reduces the intercellular communication via gap junctions. The role of Cx43 phosphorylation for gap junction dynamics, which is not only controlled by kinases but also by phosphatases [29], is reviewed in [69].

In addition to its localization at the sarcolemma, Cx43 is also present at the inner membrane of mitochondria isolated from ventricular tissue [55, 73], especially in subsarcolemmal mitochondria (SSM) and to a far lesser extent in interfibrillar mitochondria (IFM), which are located between the myofibrils [9, 75]. Compared to the detailed analysis of the phosphorylation of gap junctional Cx43, the phosphorylation of mitochondrial Cx43 is relatively unknown; however, phosphorylation at S262 and S368 has been detected [73, 76]. Within mitochondria, Cx43 influences mitochondrial respiration [7], formation of reactive oxygen species (ROS) [24, 68, 77], uptake of potassium ions [10], and opening of the mitochondrial permeability transition pore (MPTP) [20, 73].

Acute myocardial ischemia/reperfusion (**I/R**) injury elicits death of cardiomyocytes via necrosis, apoptosis, necroptosis, and pyroptosis (for review, see [26]). Both ischemic preconditioning (IPC) and ischemic postconditioning reduce myocardial infarct size after prolonged ischemia followed by reperfusion. In IPC, cycles of short, non-lethal episodes of ischemia/reperfusion (*I/R*) precedes the long-lasting ischemia, whereas in ischemic postconditioning, the short periods of *I*/*R* are applied at the onset of reperfusion following the long-lasting ischemic episode. Adenosine, bradykinin, opioids, and cytokines function as mediators or receptor-dependent triggers of cardioprotection. After a preconditioning stimulus, three major signaling pathways become activated, i.e., a) a pathway involving eNOS, protein kinase G and PKC; b) the RISK pathway (reperfusion injury salvage kinase pathway) including activation of AKT, ERK, and glycogen synthase kinase 3β; and c) the SAFE pathway (survival activating factor enhancement), in which cytokine receptors and janus-kinase/signal transducer and activator of transcription proteins are induced [25, 26]. Besides targeting the sarcoplasmic reticulum, the nucleus, as well as the sarcolemma (including gap junctions and hemichannels), mitochondria represent a common endpoint of cardioprotective maneuvers [6]. Whereas mitochondrial respiration declines after *I*/*R*, oxygen consumption is maintained after IPC. The role of ROS in the cardioprotection by IPC is complex: whereas IPC reduces the detrimental burst of ROS at the onset of reperfusion, small amounts of ROS also function as trigger molecules in the signaling cascade of IPC [66]. In addition, opening of mitochondrial ATP-dependent potassium channels is important to reduce myocardial *I*/*R* injury and the opening of these channels leads to ROS formation which activate protein kinases involved in cardioprotective signaling (for review, see [25]). Conditions favoring opening of the MPTP, which elicits mitochondrial swelling, rupture, and finally cell death, are induced by ischemia [47], and both ischemic pre- and postconditioning induce cardioprotection via the inhibition of MPTP opening at reperfusion. Also, mitochondrial dynamics, i.e., fusion and fission, are involved in *I/R* injury, whereby the inhibition of fission—and thereby a decreased fragmentation of mitochondrial networks—is effective in reducing *I*/*R* damage [49].

Cx43 is involved in myocardial *I*/*R* injury and also represents an important signaling molecule in the cardioprotection by IPC. The role of Cx43 in myocardial *I*/*R* damage comprises all aspects of Cx43 function: posttranslational modifications, protein amount, alterations of gap junction, and hemichannel opening as well as mitochondrial function [60]. Whereas ischemia is often associated with a dephosphorylation and lateralization of Cx43 at the cardiomyocyte plasma membrane [60], Cx43 phosphorylation, and localization is maintained after IPC [56, 61]. The cardioprotection by IPC depends on the presence of Cx43, since the genetic reduction of Cx43 abrogates the infarct size reduction by IPC [59, 62, 63]. Gap junctional coupling seems to be part of IPC’s cardioprotection, since blocking of Cx43-formed channels during the trigger phase abolishes the infarct size reduction of IPC [39] and IPC blocks chemical gap junctional coupling during ischemia (for review, see [46]). Plasmalemmal Cx43-formed hemichannels are predominantly closed under physiological conditions [1, 22], but they open after prolonged ischemia, which leads to cell swelling and finally cell death [65, 81]. Accordingly, the inhibition of Cx43-formed hemichannels by the peptide Gap19 reduces myocardial infarction after *I*/*R* [81] demonstrating the importance of the prevention of hemichannel opening for the reduction of myocardial damage after *I*/*R*. Since Cx43 affects mitochondrial function in several ways that influence cardioprotection [6, 12, 32], Cx43 within the the mitochondria is of special interest for myocardial *I*/*R* injury. Following *I*/*R*, phosphorylation of mitochondrial Cx43 is decreased [76]. Mitochondrial Cx43 is also involved in IPC, since a specific reduction of mitochondrial Cx43 abrogates preconditioning [55] and overexpression of mitochondrial Cx43 is sufficient to induce cell protection [43].

It is noteworthy that although ischemic postconditioning alters Cx43 phosphorylation [5, 23, 50], infarct size is effectively reduced in heterozygous Cx43-deficient mice [27]. Thus, despite its relevance for the cardioprotection by IPC, ischemic postconditioning is not dependend on Cx43.

Whereas Cx43 clearly contributes to *I/R* response, the role of Cx43 phosphorylation on the regulation of mitochondrial function, cardiomyocyte hemichannel activity, and cardioprotection by IPC is heretofore not well studied and, thus, is the goal of the present study.

## Materials and methods

### Animals

The generation of the mouse strains Cx43^MAPKmut^, Cx43^PKCmut^, and Cx43^CK1mut^ has been described previously [30, 34]. In the line Cx43^MAPKmut^, serines 255, 262, 279, and 282 (which are targeted by MAPK) were replaced by non-phosphorylatable alanine residues; in the line Cx43^PKCmut^, the PKC-targeted residue serine 368 was replaced by alanine, and in the line Cx43^CK1mut^, serines at positions 325, 328, and 330 were replaced by alanine, tyrosine, and alanine, respectively. Mutated mice were backcrossed with C57Bl6/J mice for 10 generations (Fred Hutchinson Cancer Research Center). At the University of Giessen, mutated mice were backcrossed with C57Bl6/J mice after every 6–8 generations (Cx43^MAPKmut^), after 10 generations (Cx43^PKCmut^), and after 4–6 generations (Cx43^CK1mut^). C57Bl6/J mice (Janvier, Le Genest-Saint-Isles, France) served as controls. In addition, inducible Cx43 knockout mice (Cx43^Cre−ER(T)/fl^) which express 50% of Cx43 were studied [16]. Both male and female mice of 12–22 weeks of age (18–34 g) were analyzed. Mice were kept in dark/light cycles of 12 h each and had free access to standard chow and drinking water. The present study conforms to the Guide for the Care and Use of Laboratory Animals published by the US National Institutes of Health (NIH publication No. 85–23, revised 1996) and is in accordance with the ARRIVE (Animal research: Reporting in vivo experiments) guidelines. The study was approved by the animal welfare office of the Justus-Liebig-University Giessen, Germany (522_M) and by the committee on ethical usage of animals of Ghent University (ECD16-24.)

### Isolation of subsarcolemmal mitochondria

Subsarcolemmal mitochondria (SSM) were isolated from left-ventricular (LV) tissue by differential centrifugation as already described [11] and were used to study mitochondrial function immediately after isolation. For Western Blot analysis, mitochondria were further purified by Percoll-gradient ultracentrifugation and were stored at − 80 °C. The purity of all mitochondrial preparations was verified by Western Blot analysis (Supplementary Fig. 3).

### Western Blot analysis

Protein extraction from LV tissue samples and isolated mitochondria is described in detail in the online supplement. Antibodies were used against total Cx43, Cx43 phosphorylated at S262, S325/328/330, S365, S368, and S373. Antibodies against glyceraldehyde 3-phosphate dehydrogenase (GAPDH) and manganese superoxide dismutase (MnSOD) were used for normalization of total and mitochondrial proteins, respectively.

### Mitochondrial respiration

Mitochondrial oxygen consumption was measured from 100 µg/ml mitochondrial proteins with a Clark-type electrode (Strathkelvin, Glasgow, UK) at 25 °C using glutamate and malate as substrates for complex 1 and succinate (in the presence of rotenone to inhibit complex 1) as substrate for complex 2. In addition, complex 4-mediated respiration was analyzed in the presence of *N*,*N*,*N*′,*N*′-tetramethyl-p-phenylenediamine (TMPD) and ascorbate, and uncoupling of oxidative phosphorylation was induced by the addition of FCCP (cyanide 4-(trifluoromethoxy)phenylhydrazone). Respiration was stimulated by the addition of 40 µmol/L ADP. Oxygen consumption is shown in nmol O_2_*min^−1^*mg protein^−1^ [4].

### Formation of reactive oxygen species

ROS formation was detected from 50 µg mitochondrial proteins using 50 µmol/L AmplexUltraRed. Background fluorescence without mitochondria was subtracted and the slope of the fluorescence signal was calculated for 2 min [4].

### Calcium-induced MPTP opening

The calcium retention capacities of 100 µg/ml SSM with glutamate and malate were measured at 25 °C in the presence of 0.5 µmol/L Calcium Green 5 N. Five µmol/L CaCl_2_ were added every third minute until a sudden increase in Calcium Green 5 N fluorescence occurred reflecting MPTP opening [4]. As a positive control, measurements were performed in the presence of 1 µmol/L Cyclosporine A (CsA), which inhibits MPTP opening.

### Cardiomyocyte isolation and electrophysiological recordings

The isolation of LV cardiomyocytes from wild-type (WT), Cx43^MAPKmut^, Cx43^PKCmut^, and Cx43^CK1mut^ mice was performed as previously described [42]. Mice were sacrificed by cervical dislocation. Following thoracotomy, the heart was quickly excised and transferred to a Langendorff apparatus and perfused at a constant flow (~ 3 ml/min) and temperature (37 °C). Ca^2+^-tolerant cells were used for the experiment within 6 h after isolation. Cardiomyocytes were studied using whole-cell voltage-clamp to record membrane currents. Patch-clamp experiments were conducted at room temperature (22 ± 1 °C).

### Ischemia/reperfusion in vitro

Isolated mouse hearts were Langendorff-perfused as already described [4]. The *I/R* protocol comprised of a 5 min stabilization phase, followed by 45 min ischemia and 120 min reperfusion. IPC was performed by three preceding cycles of 3 min ischemia and 5 min reperfusion. At the end of the protocol, myocardial infarction was delineated by triphenyltetrazolium chloride (TTC) staining and quantified by planimetry [4].

### Statistics

Data are presented as mean ± SEM. Western Blot data on the amount of Cx43 in total and mitochondrial protein extracts were compared by non-parametric Rank Sum test, and Western Blot data on phosphorylated Cx43, ROS formation, opening of Cx43-formed hemichannels, and sodium/calcium exchanger (NCX) parameters were analyzed by unpaired two-sided *t* test. The amount of phosphorylated and total Cx43 in isolated cardiomyocytes was compared by paired *t* test. The amount of mitochondrial protein isolated from LV tissue was compared by one-way ANOVA. Data on heart weight and heart weight/body weight, mitochondrial respiration, calcium-induced MPTP opening, myocardial infarction, end-diastolic pressure (EDP), and left-ventricular developed pressure (LVDP) were compared by two-way ANOVA followed by Holm–Sidak test**.** Analyses were done with SigmaStat 3.5 (Systat, Software GmbH, Erkrath, Germany) and OriginLab software (OriginLab Corporation, USA). A *p* value < 0.05 was considered to indicate a significant difference.

A detailed description of the Methods is given in the online supplement.

## Results

The amount of Cx43 was measured by Western Blot analysis in total LV protein extracts of WT, Cx43^MAPKmut^, Cx43^PKCmut^, and Cx43^CK1mut^ mice, respectively (Table 1). Whereas Cx43 was moderately reduced in Cx43^MAPKmut^ mice, a more severe reduction of the protein was detected in Cx43^CK1mut^ mice and especially in Cx43^PKCmut^ mice (all strains *p* < 0.05). The phosphorylation of Cx43 was analyzed by Western blot at S262, S325/328/330, S365, S368, and S373. Antibodies against Cx43 phosphorylated at S255, S279, and S282 could not be validated in initial Western Blot analyses; therefore, the Cx43 phosphorylation status at residues targeted by MAPK was based on S262 only. In total LV protein extracts of Cx43^MAPKmut^ mice, the ratio of phosphorylated over total Cx43 was not altered. In Cx43^PKCmut^ and Cx43^CK1mut^ mice, Cx43 phosphorylation was significantly decreased at S365, but increased at S262. Additionally, Cx43 phosphorylation was diminished at S325/328/330 and S373 in Cx43^PKCmut^ and at S368 in Cx43^CK1mut^ mice. Treatment of isolated cardiomyocytes from wild-type mice for 2 h or 6 h with inhibitors against PKC (GF109203X) or CK1 (CKI-7) did not increase Cx43 S262 phosphorylation or reduce Cx43 expression as in Cx43^PKCmut^ or Cx43^CK1mut^ mice (Supplementary Fig. 1). As expected, no immunoreactivity was detected using antibodies against S262 in Cx43^MAPKmut^, against S368 in Cx43^PKCmut^, and against S325/328/330 in Cx43^CK1mut^ mice, which proves the specificity of the antibodies and the genotype of the mice (Supplementary Fig. 2).

**Table 1:** Phosphorylation of Cx43 in LV and SSM samples of Cx43^MAPKmut^, Cx43^PKCmut^, and Cx43^CK1mut^ mice

Strain	Sample	P-S262/Cx43 (a.u.)	P-S325/328/330/ Cx43 (a.u.)	P-S365/Cx43 (a.u.)	P-S368/Cx43 (a.u.)	P-S373/Cx43 (a.u.)	Cx43 in % WT
WT Cx43^MAPKmut^	LV	n.d	0.51 ± 0.08, *n* = 8 0.54 ± 0.08, *n* = 8 *p* = ns	0.17 ± 0.04, *n* = 8 0.15 ± 0.07, *n* = 8 *p* = ns	0.53 ± 0.04, *n* = 8 0.41 ± 0.03, *n* = 7, *p* = 0.06	0.62 ± 0.15, *n* = 8 0.41 ± 0.05, *n* = 7, *p* = ns	100 ± 15,5, *n* = 8 84.6 ± 6.9, *n* = 8 *p* < 0.05
WT Cx43^PKCmut^	LV	0.55 ± 0.08, *n* = 6 2.16 ± 0.24, *n* = 6 *p* < 0.05	1.01 ± 0.1, *n* = 8 0.24 ± 0.03, *n* = 8 *p* < 0.05	0.35 ± 0.12, *n* = 8 0.02 ± 0.002, *n* = 8 *p* < 0.05	n.d	0.58 ± 0.12, *n* = 6 0.15 ± 0.02, *n* = 6 *p* < 0.05	100 ± 10.0, *n* = 8 38.9 ± 4.4, *n* = 8 *p* < 0.05
WT Cx43^CK1mut^	LV	0.26 ± 0.06, *n* = 6 0.71 ± 0.15, *n* = 6 *p* < 0.05	n.d	0.87 ± 0.09, *n* = 6 0.50 ± 0.05, *n* = 6 *p* < 0.05	0.72 ± 0.03, *n* = 6 0.56 ± 0.06, *n* = 6 *p* < 0.05	0.62 ± 0.09, *n* = 6 0.41 ± 0.06, *n* = 6 *p* = ns	100 ± 13.6, *n* = 6 53.5 ± 7.1, *n* = 6 *p* < 0.05
WT Cx43^MAPKmut^	SSM	n.d	0.42 ± 0.04, *n* = 7 0.30 ± 0.02, *n* = 10 *p* < 0.05	0.17 ± 0.02, *n* = 7 0.20 ± 0.03, *n* = 9 *p* = ns	0.87 ± 0.03, *n* = 8 0.98 ± 0.05, *n* = 11 *p* = ns	0.19 ± 0.02, *n* = 8 0.08 ± 0.01, *n* = 9 *p* < 0.05	100 ± 14.4, *n* = 8 73.2 ± 10.0, *n* = 11 *p* < 0.05
WT Cx43^PKCmut^	SSM	0.22 ± 0.03, *n* = 8 0.35 ± 0.04, *n* = 8 *p* < 0.05	0.025 ± 0.003, *n* = 6 0.015 ± 0.003, *n* = 6 *p* < 0.05	0.38 ± 0.02, *n* = 8 0.87 ± 0.13, *n* = 8 *p* < 0.05	n.d	0.19 ± 0.02, *n* = 8 0.53 ± 0.05, *n* = 5 *p* < 0.05	100 ± 15.1, *n* = 6 9.1 ± 2.1, *n* = 6 *p* < 0.05
WT Cx43^CK1mut^	SSM	0.14 ± 0.02, *n* = 6 0.49 ± 0.11, *n* = 6 *p* < 0.05	n.d	0.62 ± 0.08, *n* = 6 0.57 ± 0.05, *n* = 6 *p* = ns	0.52 ± 0.08, *n* = 6 0.49 ± 0.04, *n* = 5 *p* = ns	0.18 ± 0.05, *n* = 6 0.17 ± 0.08, *n* = 6 *p* = ns	100 ± 10.8, *n* = 6 61.5 ± 3.9, *n* = 5 *p* < 0.05

Western blot analysis was performed for Cx43 phosphorylated at S262, S325/328/330, S365, S368, or S373 on left-ventricular (LV) and subsarcolemmal mitochondria (SSM) protein samples isolated from wild-type mice (WT) or Cx43^MAPKmut^, Cx43^PKCmut^, and Cx43^CK1mut^ mice. Phosphorylated Cx43 was normalized to total Cx43 and signal intensities are shown in arbitrary units (a.u.). Total Cx43 was normalized to GAPDH in LV and to MnSOD in SSM protein samples and signal intensities from Cx43^MAPKmut^, Cx43^PKCmut^ or Cx43^CK1mut^ mice are shown in percentage of that in WT samples; n.d.: not determined, ns: not significant

SSM were isolated from LV tissue of WT, Cx43^MAPKmut^, Cx43^PKCmut^, and Cx43^CK1mut^ mice, and the amount of isolated proteins per mg LV tissue was quantified. An exemplary Western Blot demonstrating the purity of mitochondrial preparations is demonstrated in Supplementary Fig. 3. A trend towards reduced amounts of isolated SSM was found in Cx43^CK1mut^ mice; however, there was no significant difference (amount of isolated SSM in µg protein/mg LV tissue: WT: 2.34 ± 0.18, n = 8; Cx43^MAPK^: 1.72 ± 0.42, *n* = 5; Cx43^PKCmut^: 2.13 ± 0.25, *n* = 5; Cx43^CK1mut^: 1.50 ± 0.17, *n* = 6, *p* = ns). Western Blot analysis was performed to characterize the amount of Cx43 in SSM isolated from LV tissue of WT, Cx43^MAPKmut^, Cx43^PKCmut^, and Cx43^CK1mut^ mice (Table 1). Compared to total LV proteins, similar reductions of mitochondrial Cx43 were detected in Cx43^MAPKmut^ and Cx43^CK1mut^, whereas a further decrease to 9 ± 2% was measured in Cx43^PKCmut^ SSM (all strains *p* < 0.05). The analysis of the phosphorylation status of mitochondrial Cx43 displayed decreased phosphorylation at S325/328/330 and S373 in Cx43^MAPKmut^ mice. In mitochondria from Cx43^PKCmut^ mice, the Cx43 phosphorylation at S325/328/330 was reduced, but it was increased at S262, S365, and S373. In SSM isolated from Cx43^CK1mut^ mice, S262 was increasingly phosphorylated compared to WT SSM. Original Western blots and single values of the data presented in Table 1 are shown in the online supplement (Supplementary Figs. 4 –11).

Since the relative phosphorylation of Cx43 and also the absolute amount of the phosphorylated protein may be important, Western blot data of phosphorylated Cx43 were also normalized to the housekeeping proteins GAPDH (LV proteins) or MnSOD (SSM proteins, Table 2). Due to the low amount of Cx43 in the Cx43^PKCmut^ mitochondria, the amount of Cx43 phosphorylated at S262 and S365 was reduced when normalized to MnSOD, whereas it was increased when normalized to total Cx43. The enhanced phosphorylation of S262 in Cx43^PKCmut^ and Cx43^CK1mut^ as seen by normalization to total Cx43 was not detected by normalization to GAPDH or MnSOD.

**Table 2:** Phosphorylation of Cx43 in LV and SSM samples of Cx43^MAPKmut^, Cx43^PKCmut^, and Cx43^CK1mut^ mice

Strain	Sample	P-S262 (a.u.)	P-S325/328/330 (a.u.)	P-S365 (a.u.)	P-S368 (a.u.)	P-S373 (a.u.)
WT Cx43^MAPKmut^	LV	n.d	1.64 ± 0.60, *n* = 8 1.18 ± 0.23, *n* = 8 *p* = ns	0.48 ± 0.15 *n* = 8 0.35 ± 0.09, *n* = 8 *p* = ns	1.17 ± 0.26, *n* = 8 0.70 ± 0.0, *n* = 7, *p* = ns	0.57 ± 0.17, *n* = 8 0.35 ± 0.07, *n* = 7, *p* = ns
WT Cx43^PKCmut^	LV	0.74 ± 0.07, *n* = 6 0.90 ± 0.22, *n* = 6 *p* = ns	1.26 ± 0.18, *n* = 8 0.12 ± 0.03, *n* = 8 *p* < 0.05	0.76 ± 0.34, *n* = 8 0.02 ± 0.002, *n* = 8 *p* < 0.05	n.d	0.60 ± 0.13, *n* = 6 0.07 ± 0.02, *n* = 6 *p* < 0.05
WT Cx43^CK1mut^	LV	0.27 ± 0.05, *n* = 6 0.48 ± 0.18, *n* = 6 *p* = ns	n.d	0.88 ± 0.13, *n* = 6 0.24 ± 0.04, *n* = 6 *p* < 0.05	0.92 ± 0.05, *n* = 6 0.40 ± 0.06, *n* = 6 *p* < 0.05	0.87 ± 0.20, *n* = 6 0.31 ± 0.04, *n* = 6 *p* < 0.05
WT Cx43^MAPKmut^	SSM	n.d	0.53 ± 0.06, *n* = 7 0.45 ± 0.06, *n* = 10 *p* = ns	0.19 ± 0.04, *n* = 7 0.19 ± 0.04, *n* = 9 *p* = ns	0.66 ± 0.11, *n* = 8 0.63 ± 0.06, *n* = 11 *p* = ns	0.18 ± 0.02, *n* = 8 0.07 ± 0.02, *n* = 9 *p* < 0.05
WT Cx43^PKCmut^	SSM	0.11 ± 0.0, *n* = 8 0.04 ± 0.005, *n* = 8 *p* < 0.05	0.50 ± 0.03, *n* = 6 0.03 ± 0.005, *n* = 6 *p* < 0.05	0.35 ± 0.04, *n* = 8 0.08 ± 0.01, *n* = 8 *p* < 0.05	n.d	0.11 ± 0.02, *n* = 8 0.02 ± 0.004, *n* = 5 *p* < 0.05
WT Cx43^CK1mut^	SSM	0.08 ± 0.01, *n* = 6 0.22 ± 0.08 *n* = 6 *p* = ns	n.d	0.46 ± 0.11, *n* = 6 0.31 ± 0.04, *n* = 6 *p* = ns	0.56 ± 0.11, *n* = 6 0.33 ± 0.05, *n* = 5 *p* = ns	0.13 ± 0.02, *n* = 6 0.10 ± 0.03, *n* = 6 *p* = ns

Western blot analysis was performed for Cx43 phosphorylated at S262, S325/328/330, S365, S368, and S373 on left-ventricular (LV) and subsarcolemmal mitochondria (SSM) protein samples isolated from wild-type mice (WT) or Cx43^MAPKmut^, Cx43^PKCmut^, and Cx43^CK1mut^ mice. Signal intensities are shown in arbitrary units (a.u.). Phosphorylated Cx43 was normalized to GAPDH in LV samples and to MnSOD in SSM samples, n.d.: not determined, ns: not significant

**Fig. 1:**
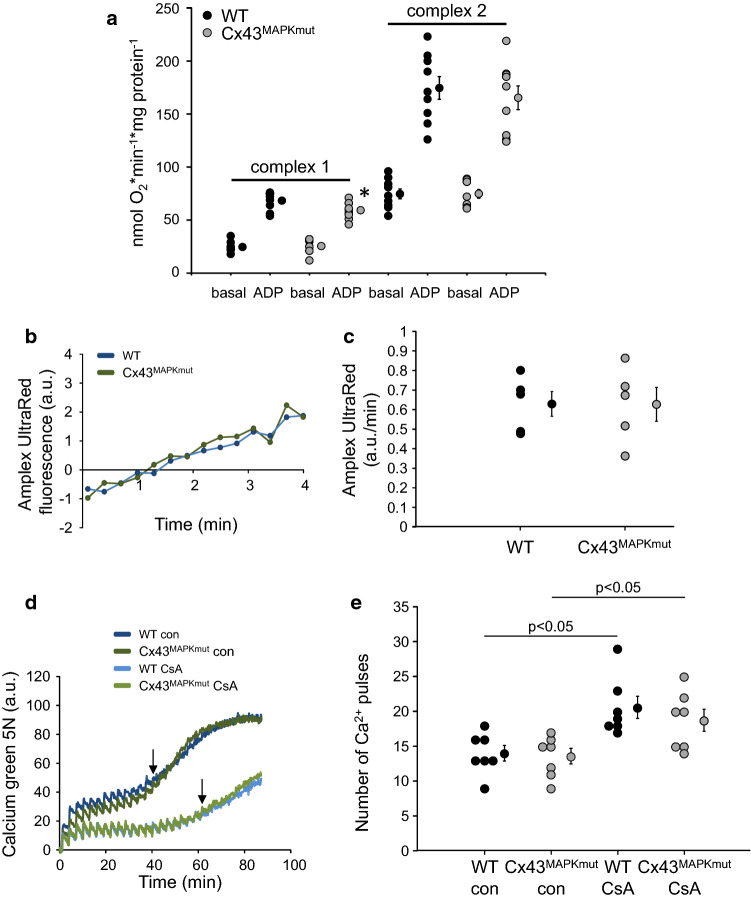
Mitochondrial function in Cx43^MAPKmut^ mice. **a **Mitochondrial oxygen consumption of SSM isolated from wild-type (WT) and Cx43^MAPKmut^ mice respiring on complex 1 or complex 2 substrates without (basal) and with ADP to stimulate respiration. WT: *n* = 9, Cx43^MAPKmut^ *n* = 9, *: *p* < 0.05 vs ADP WT as analyzed by two-way ANOVA. **b **Original traces showing ROS formation detected by Amplex UltraRed fluorescence in arbitrary units (a.u.) from WT and Cx43^MAPKmut^ SSM. **c **Quantification of ROS formation (slope of Amplex UltraRed fluorescence in a.u./min) from WT (*n* = 5) and Cx43^MAPKmut^ SSM (*n* = 5), *p* = ns as analyzed by unpaired *t* test. **d **Original traces showing Calcium green 5 N fluorescence (in a.u.) of SSM isolated from WT and Cx43^MAPKmut^ mice. Every third minute, 5 µmol/L CaCl_2_ was added until a sudden increase in Calcium green 5 N fluorescence occurred (↓) which reflects MPTP opening. Measurements were performed without (con) and with Cyclosporine A (CsA), which was used as a positive control to inhibit MPTP opening. **e **The number of calcium pulses which was necessary to induce MPTP opening was calculated from SSM of WT and Cx43^MAPKmut^ mice without (con) and with CsA. WT (*n* = 7), Cx43^MAPKmut^ (*n* = 7). Data were compared by two-way ANOVA

**Fig. 2:**
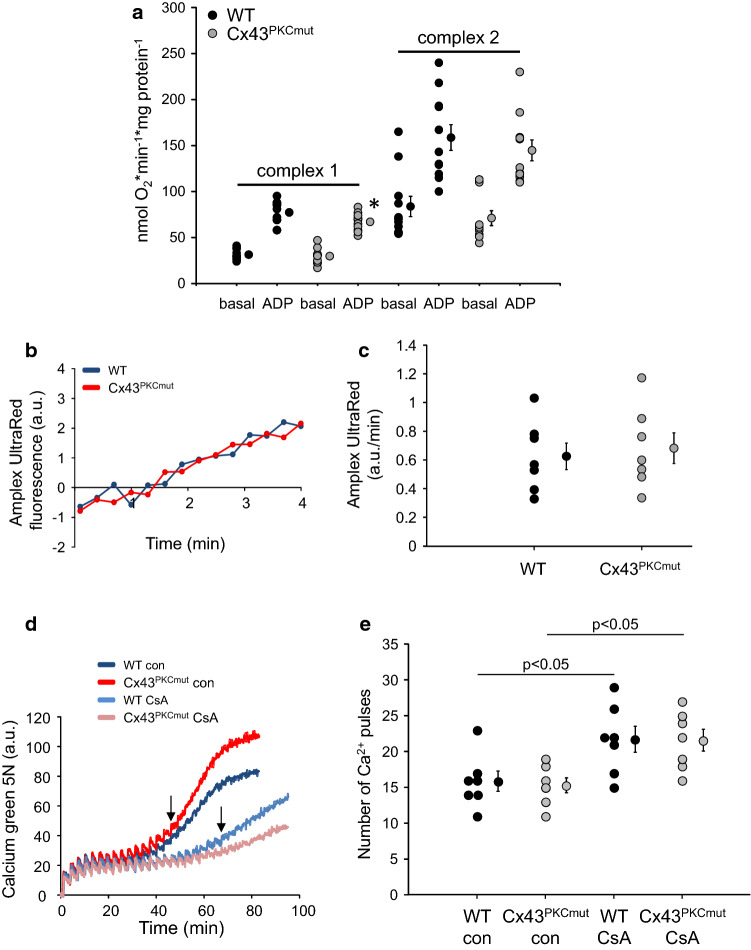
Mitochondrial function in Cx43^PKCmut^ mice. **a** Mitochondrial oxygen consumption of SSM isolated from wild-type (WT) and Cx43^PKCmut^ mice respiring on complex 1 or complex 2 substrates without (basal) and with ADP to stimulate respiration. WT: *n* = 11, Cx43^PKCmut^ *n* = 11, *p < 0.05 vs ADP WT as analyzed by two-way ANOVA. **b** Original traces showing ROS formation detected by Amplex UltraRed fluorescence in arbitrary units (a.u.) from WT and Cx43^PKCmut^ SSM. **c** Quantification of ROS formation (slope of Amplex UltraRed fluorescence in a.u./min) from WT (*n* = 7) and Cx43^PKCmut^ SSM (*n* = 7), *p* = ns, data were compared by unpaired *t*-test. **d** Original traces showing Calcium green 5 N fluorescence (in a.u.) of SSM isolated from WT and Cx43^PKCmut^ mice. Every third minute, 5 µmol/L CaCl_2_ was added until a sudden increase in Calcium green 5 N fluorescence occurred (↓) which reflects MPTP opening. Measurements were performed without (con) and with Cyclosporine A (CsA), which was used as a positive control to inhibit MPTP opening. **e** The number of calcium pulses which was necessary to induce MPTP opening was calculated from SSM of WT and Cx43^PKCmut^ mice without (con) and with CsA. WT (*n* = 7), Cx43^PKCmut^ (*n* = 7). Data were compared by two-way ANOVA

**Fig. 3:**
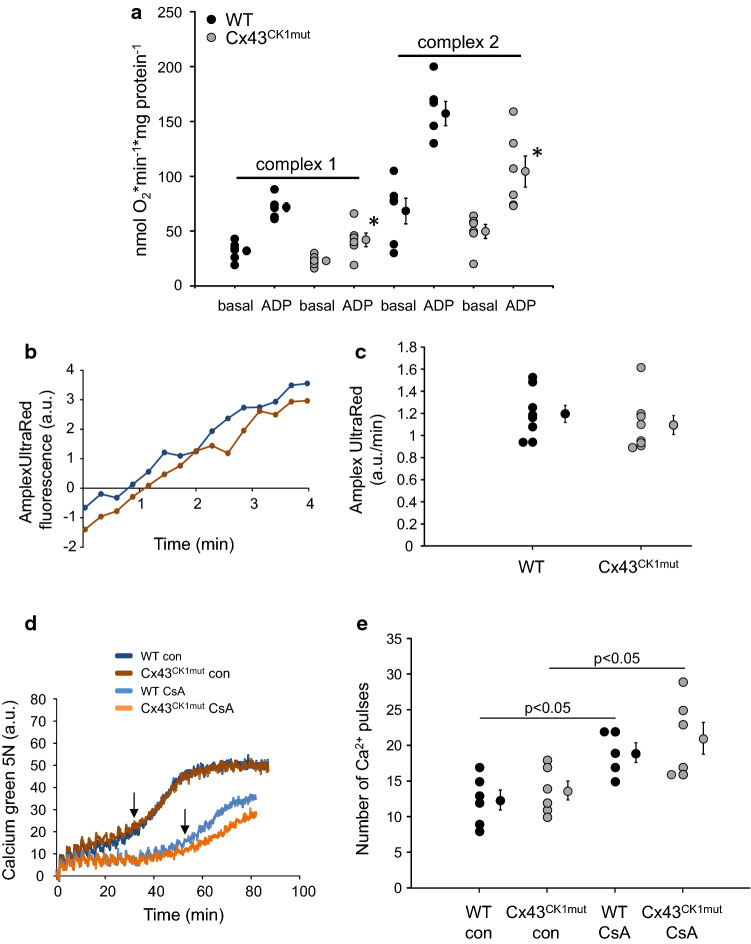
Mitochondrial function in Cx43^CK1mut^ mice. **a** Mitochondrial oxygen consumption of SSM isolated from wild-type (WT) and Cx43^CK1mut^ mice respiring on complex 1 or complex 2 substrates without (basal) and with ADP to stimulate respiration. WT: *n* = 6, Cx43^CK1mut^ *n* = 6, *p < 0.05 vs ADP WT as analyzed by two-way ANOVA. **b** Original traces showing ROS formation detected by Amplex UltraRed fluorescence in arbitrary units (a.u.) from WT and Cx43^CK1mut^ SSM. **c** Quantification of ROS formation (slope of Amplex UltraRed fluorescence in a.u./min) from WT (*n* = 8) and Cx43^CK1mut^ SSM (*n* = 8), *p* = ns, data were compared by unpaired t test. **d** Original traces showing calcium green 5 N fluorescence (in a.u.) of SSM isolated from WT and Cx43^CK1mut^ mice. Every third minute, 5 µmol/L CaCl_2_ was added until a sudden increase in Calcium green 5 N fluorescence occurred (↓) which reflects MPTP opening. Measurements were performed without (con) and with Cyclosporine A (CsA), which was used as a positive control to inhibit MPTP opening. **e** The number of calcium pulses which was necessary to induce MPTP opening was calculated from SSM of WT and Cx43^CK1mut^ mice without (con) and with CsA. WT (con: *n* = 6; CsA: *n* = 5) and Cx43^CK1mut^ (*n* = 6). Data were compared by two-way ANOVA

To investigate whether or not mutations at MAPK, PKC, and CK1 target sites within Cx43 influence mitochondrial function, respiration, ROS formation and MPTP opening were measured in SSM isolated from WT, Cx43^MAPKmut^ (Fig. 1), Cx43^PKCmut^ (Fig. 2), and Cx43^CK1mut^ mice (Fig. 3). Whereas basal respiration (both complex 1 und 2 substrates) was not affected by mutating Cx43 phosphorylation sites, ADP-stimulated oxygen consumption of SSM respiring on complex 1 substrates was decreased in SSM from the three analyzed mouse strains, with the highest reduction in SSM of Cx43^CK1mut^ mice. Using complex 2 substrates, ADP-stimulated respiration was diminished in Cx43^CK1mut^ SSM only. Despite a trend towards a reduced respiratory control rate (RCR; ADP-stimulated respiration/basal respiration) in Cx43^CK1mut^ SSM, there was no significant difference in the RCR between SSM isolated from WT mice and mice with mutated Cx43 phosphorylation sites. There was a trend towards a reduced oxygen consumption driven by *N*,*N*,*N*′,*N*′-tetramethyl-p-phenylenediamine (TMPD)/ascorbate (complex 4 respiration) and a significantly decreased uncoupled respiration using carbonyl cyanide 4-(trifluoromethoxy)phenylhydrazone (FCCP) in Cx43^CK1mut^ compared to WT SSM (Table 3). The protein amounts of complexes 1–5 of the electron transport chain were not altered in Cx43^MAPKmut^ and Cx43^PKCmut^, whereas the level of complex 1 was significantly enhanced in Cx43^CK1mut^ mice (Supplementary Fig. 12). ROS formation was similar between SSM isolated from WT, Cx43^MAPKmut^, Cx43^PKCmut^, and Cx43^CK1mut^ mice. In addition, the analysis of calcium-induced MPTP opening revealed no differences between the analyzed mouse lines. The use of cyclosporine A (CsA) as an inhibitor of MPTP opening significantly enhanced the calcium retention capacity in all analyzed strains.

**Table 3:** Mitochondrial oxygen consumption in SSM isolated from WT, Cx43^MAPKmut^, Cx43^PKCmut^, and Cx43^CK1mut^ mice

	WT (*n* = 9)	Cx43^MAPKmut^ (*n* = 9)	WT (*n* = 11)	Cx43^PKCmut^ (*n* = 11)	WT (*n* = 6)	Cx43^CK1mut^ (*n* = 6)
RCR complex 1	2.83 ± 0.16	2.56 ± 0.33	2.50 ± 0.14	2.38 ± 0.19	2.39 ± 0.32	1.80 ± 0.15
RCR complex 2	2.40 ± 0.20	2.21 ± 0.11	2.02 ± 0.17	2.20 ± 0.23	2.86 ± 0.70	2.24 ± 0.33
TMPD/ ascorbate	536 ± 35	485 ± 35	437 ± 39	409 ± 35	443 ± 55	301 ± 39 (*p* = 0.06)
FCCP	698 ± 44	632 ± 33	634 ± 55	620 ± 58	687 ± 68	457 ± 44*

SSM were isolated from the left ventricle (LV) of wild-type (WT), Cx43^MAPKmut^, Cx43^PKCmut^, and Cx43^CK1mut^ mice and basal and ADP-stimulated respiration were measured. The respiratory control rate (RCR) is shown as the ADP-stimulated respiration divided by the basal respiration using substrates for complex 1 or complex 2. Mitochondrial respiration (in nmolO_2_*min^−1^* mg protein^−1^) was analyzed in the presence of N,N,N′,N′-tetramethyl-p-phenylenediamine (TMPD)/ascorbate (0.27 or 0.82 mM, respectively) or carbonyl cyanide 4-(trifluoromethoxy)phenylhydrazone (FCCP, 100 nM). Data are presented for the SSM isolated from mice with mutated Cx43 phosphorylation sites and for the respective WT controls

*p < 0.05 vs WT, unpaired *t* test

**Fig.4:**
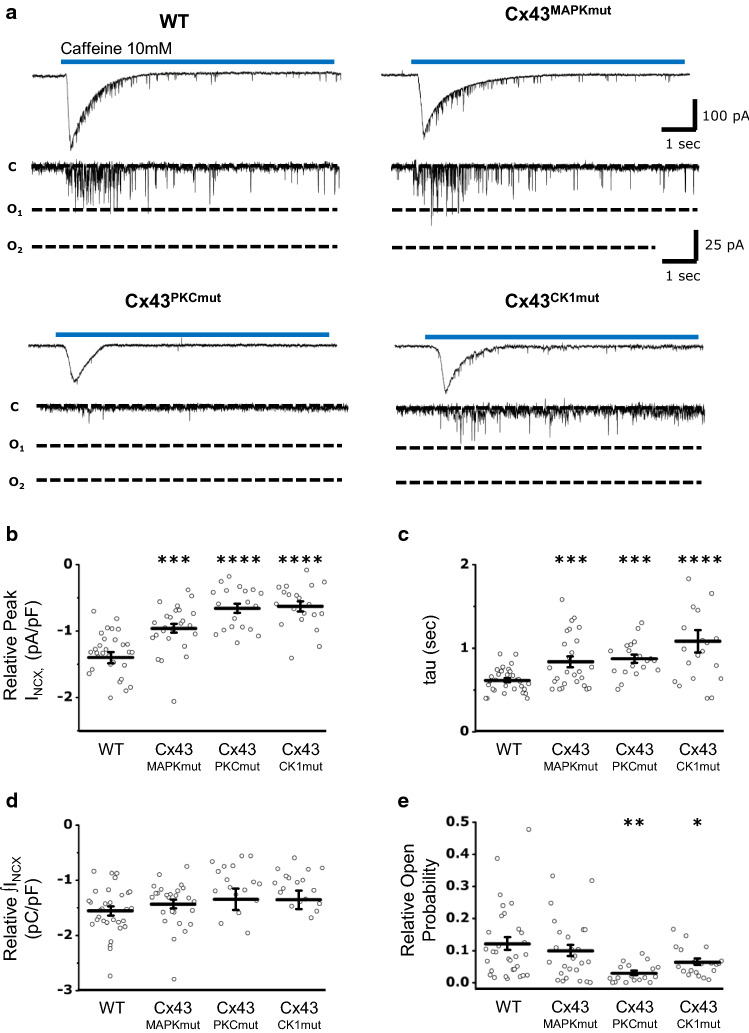
Effect of altered Cx43 phosphorylation on hemichannel gating. **a** Representative sodium/calcium exchanger (NCX) current traces with superimposed Cx43 unitary current events triggered by caffeine (10 mmol/L) at *V*_h_ = − 70 mV. **b** Average data showing significantly reduced normalized NCX peak amplitudes in Cx43^MAPKmut^, Cx43^PKCmut^, and Cx43^CK1mut^ cardiomyocytes compared to wild-type (WT) cells. NCX currents were normalized on the cell capacity to minimize the difference due to cell size. **c** Summary data illustrating the time constant of the recovery phase in cardiomyocytes of WT, Cx43^MAPKmut^, Cx43^PKCmut^, and Cx43^CK1mut^ mice. **d** Overall sarcoplasmic reticulum calcium equilibrium. **e** Quantification of the Cx43 hemichannel open probability demonstrating reduced single-channel activity in Cx43^PKCmut^ and Cx43^CK1mut^ mice compared to control conditions. WT (*N* = 6; *n* = 31), (Cx43^MAPKmut^ (*N* = 9; *n* = 26), Cx43^PKCmut^ (*N* = 3; *n* = 19), Cx43^CK1mut^ (*N* = 4; *n* = 20),). *p < 0.05; **p < 0.01; ***p < 0.001; ****p < 0.0001 (unpaired Student’s t test)

Cx43 hemichannel activity was compared between WT, Cx43^MAPKmut^, Cx43^PKCmut^, and Cx43^CK1mut^ mice using patch-clamp experiments. LV cardiomyocytes were voltage-clamped at the resting potential of − 70 mV. Short caffeine applications were used to trigger Cx43 hemichannel activity, which is characterized by spiking unitary events superimposed on the macroscopic sodium/calcium exchanger (NCX) current [42] (Fig. 4a). Altering Cx43 phosphorylation on the MAPK, PKC, and CK1 target sites significantly reduced the amplitude and slowed down the kinetics of the NCX current compared to control (Fig. 4b, c). However, the changes in NCX transporter kinetics did not reflect an alteration of the subsarcolemmal calcium content (Fig. 4d), for which NCX is considered to be a valuable indicator [79]. Cx43 hemichannel activity was significantly reduced in Cx43^CK1mut^ and in Cx43^PKCmut^ mice, but not in Cx43^MAPKmut^ mice (Fig. 4e).

**Fig. 5:**
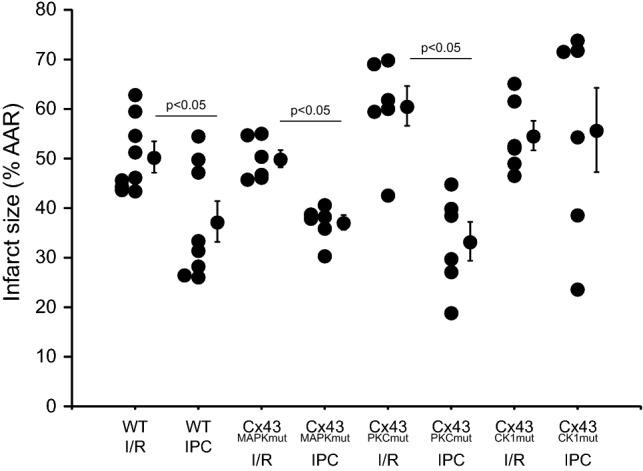
Influence of Cx43 phosphorylation on the cardioprotection by IPC in vitro. Infarct sizes (in % of the area at risk) are shown for wild-type (WT), Cx43^MAPKmut^, Cx43^PKCmut^, and Cx43^CK1mut^ mice subjected to ischemia/reperfusion (*I*/*R*) or ischemic preconditioning (IPC) in vitro. In addition to the individual infarct sizes of each animal, the mean value ± SEM of each group is presented, WT (*I*/*R*: *n* = 9, IPC: *n* = 8), Cx43^MAPKmut^ (*I*/*R*: *n* = 6, IPC; *n* = 6), Cx43^PKCmut^ (*I*/*R*: *n* = 6, IPC; *n* = 6), Cx43^CK1mut^ (*I*/*R*: *n* = 6, IPC; *n* = 6), data were compared by two-way ANOVA

To characterize the importance of Cx43 phosphorylation for myocardial *I/R* injury, isolated hearts were Langendorff-perfused and underwent a protocol of *I/R* (45 min ischemia and 120 min reperfusion) without and with IPC. Heart rate returned to 600 bpm in all groups within 10 min reperfusion and hemodynamic data are shown in Table 4. Hearts of WT, Cx43^MAPKmut^, Cx43^PKCmut^, and Cx43^CK1mut^ mice revealed similar infarct sizes after *I/R* (Fig. 5). Following IPC, infarct size was reduced in WT, Cx43^MAPKmut^ and Cx43^PKCmut^ hearts, whereas IPC did not protect hearts of Cx43^CK1mut^ mice. In Cx43^Cre−ER(T)/fl^ hearts, which express 50% of Cx43 (an amount comparable to that in Cx43^CK1mut^ mice), infarct size after *I/R* with IPC was 77.8 ± 7.4% of the area at risk (*n* = 4, *p* = ns vs. WT), thereby indicating a loss of cardioprotection by IPC also in this strain.

**Table 4:** Summary of the baseline parameters and hemodynamic data throughout ischemia–reperfusion protocols in vitro

Genotype	Protocol	*n* value	Body weight (g)	Heart weight/body weight (mg/g)	EDP (mm Hg)				LVDP (mm Hg)		
					Basal	End of ischemia	10 min reperfusion	End of reperfusion	Basal	10 min reperfusion	End of reperfusion
WT	IR in vitro	9	27.3 ± 1.5	6.38 ± 0.22	12.3 ± 0.4	65.2 ± 1.9	52.2 ± 4.7*	28.1 ± 3.3*	103.8 ± 3.6	58.7 ± 9.2	54.2 ± 2.7
WT	IPC in vitro	8	26.9 ± 1.5	6.74 ± 0.31	11.4 ± 0.7	63.0 ± 7.1	27.0 ± 2.6	16.2 ± 2.1	102.6 ± 6.8	66.3 ± 3.8	61.1 ± 5.8
Cx43^MAPKmut^	IR in vitro	6	23.0 ± 1.3^+^	5.96 ± 0.15	13.2 ± 0.6	64.6 ± 1.1	45.1 ± 4.0*	24.0 ± 1.1	88.9 ± 6.0	48.2 ± 8.8	44.7 ± 3.9
Cx43^MAPKmut^	IPC in vitro	6	22.7 ± 1.2	6.40 ± 0.28	13.7 ± 0.9^#^	53.3 ± 4.7	30.6 ± 2.9	19.5 ± 2.4	90.2 ± 5.5	59.9 ± 4.4	45.3 ± 2.3^#^
Cx43^PKCmut^	IR in vitro	6	25.3 ± 0.8	6.17 ± 0.42	11.7 ± 0.7	51.6 ± 7.2	44.1 ± 4.5*	22.5 ± 3.4	96.1 ± 10.1	48.9 ± 8.5	42.1 ± 6.2*
Cx43^PKCmut^	IPC in vitro	6	25.7 ± 1.4	6.29 ± 0.22	13.1 ± 0.8	52.0 ± 5.4	32.0 ± 4.6	17.8 ± 2.3	100.2 ± 8.5	64.9 ± 4.1	58.5 ± 5.0
Cx43^CK1mut^	IR in vitro	6	26.3 ± 1.4	6.48 ± 0.22	11.5 ± 1.0	69.5 ± 5.3	47.7 ± 5.3	26.7 ± 3.1	97.3 ± 5.7	45.9 ± 7.6	52.5 ± 3.1
Cx43^CK1mut^	IPC in vitro	6	24.7 ± 1.9	6.30 ± 0.21	13.0 ± 1.0	59.7 ± 4.9	46.3 ± 5.3^#^	29.8 ± 2.8^#^	93.1 ± 10.7	46.6 ± 5.0	48.9 ± 4.0

End-diastolic pressure (EDP) and left-ventricular developed pressure (LVDP) in wild-type (WT) and Cx43^MAPKmut^, Cx43^PKCmut^, and Cx43^CK1mut^ hearts undergoing *I*/*R* without and with ischemic preconditioning (IPC). Basal data were collected at the end of the stabilization period. *p < 0.05 *I*/*R* vs IPC of the same genotype, # *p *< 0.05 vs IPC WT, + *p* < 0.05 vs I/R WT

## Discussion

The present study demonstrates that the specific phosphorylation of Cx43 at MAPK, PKC, or CK1 target sites influenced the phosphorylation and the amount of Cx43 in LV and mitochondrial protein extracts. The respective mutations in the three mouse lines altered mitochondrial oxygen consumption without effects on ROS formation and MPTP opening. Hemichannel activity was reduced in cardiomyocytes of Cx43^PKCmut^ and Cx43^CK1mut^ mice, whereas the cardioprotection by IPC was abrogated in Cx43^CK1mut^ mice only.

At first, we analyzed the expression and phosphorylation of Cx43 in total LV proteins extracted from Cx43^MAPKmut^, Cx43^PKCmut^, and Cx43^CK1mut^ mice. Compared to WT proteins, we detected a moderate decrease in total Cx43 in Cx43^MAPKmut^ mice. The analysis of the amount of Cx43 in this strain in vascular smooth muscle cells did not demonstrate reduced levels of Cx43, which may be due to the different tissue/cell types analyzed [34]. In Cx43^PKCmut^ and Cx43^CK1mut^ mice, we found a similar decrease of Cx43 in mouse hearts as previously published [30, 54, 70]. Whereas others suggested that the decreased Cx43 protein levels in Cx43^CK1mut^ mice are due to posttranscriptional alterations [70], the reason for the reduced Cx43 protein amount in Cx43^MAPKmut^, Cx43^PKCmut^, and Cx43^CK1mut^ mice remains unclear. Pharmacological inhibition of PKC or CK1 did not reduce Cx43 expression as in Cx43^PKCmut^ and Cx43^CK1mut^ mice, indicating that those interventions are not able to mimic the complex situation in vivo, where reduced gap junctional stability and Cx43 down-regulation are observed [54, 67, 70]. A failure to reduce Cx43 phosphorylation and amount of the whole-cell protein under baseline conditions using PKC and CK1 inhibitors has already been shown in different cell types, including cardiomyocytes [13, 15, 58, 69, 78, 82].

We addressed the question whether the mutation of phosphorylation sites within Cx43 influences the phosphorylation status at other serine residues. The analyses of LV total proteins of Cx43^PKCmut^ and Cx43^CK1mut^ mice demonstrated alterations of Cx43 phosphorylation at all residues analyzed, whereas such distinct phosphorylation was not observed in Cx43^MAPKmut^ mice. Our data confirm a previous study showing enhanced phosphorylation of Cx43 at S262 in Cx43^CK1mut^ mice [70]. Therefore, mutations of S325/328/330 or S368 are sufficient to induce significant changes in Cx43 phosphorylation at multiple residues including both enhanced and reduced phosphorylation at other amino acids. Possibly, the phosphorylation status of Cx43 has an impact on the binding of kinases or phosphatases and may thereby effect the phosphorylation of other target proteins [70].

In Cx43^MAPKmut^ and Cx43^CK1mut^ mice, we found comparable reductions of Cx43 in total and in mitochondrial protein extracts, whereas in Cx43^PKCmut^ mice, Cx43 was further reduced. Thus, the phosphorylation of Cx43 at S368 is important for the mitochondrial amount of Cx43. Yet, the mechanisms that prevent a mitochondrial translocation of Cx43 not phosphorylated at S368 remain unclear.

Previous studies indicated a phosphorylation of mitochondrial Cx43 at S262 [73] and S368 [64, 73] and these findings are confirmed in the present analysis. Additionally, we detected a phosphorylation of mitochondrial Cx43 at S325/328/330, S365, and S373, which demonstrates that under physiological conditions, the mitochondrial fraction of the protein is phosphorylated at multiple residues.

The pattern of Cx43 phosphorylation differed between total LV and mitochondrial proteins in Cx43^MAPKmut^, Cx43^PKCmut^, and Cx43^CK1mut^ mice. We cannot judge from the present data if only Cx43 with a specific phosphorylation pattern is imported into the mitochondria, or if the phosphorylation status of the protein is achieved within the organelle. Since several kinases and phosphatases have been detected in mitochondria [41], the latter is also possible.

We addressed the question whether the mutation of Cx43 phosphorylation sites alters mitochondrial function. Compared to wild-type mitochondria, mitochondria from Cx43^MAPKmut^ and Cx43^PKCmut^ mice revealed a moderate, but significant reduction of ADP-stimulated oxygen consumption when respiring on complex 1 substrates, whereas mitochondria from Cx43^CK1mut^ showed decreased oxygen consumption when respiring on complex 1 and complex 2 substrates. Also, the uncoupled respiration in the presence of FCCP, which indicates the maximum rate of respiration that can be achieved within the mitochondrial preparation, was diminished in Cx43^CK1mut^ mitochondria and there was also a trend towards a reduced TMPD/ascorbate-stimulated (complex 4) respiration. Therefore, the mutation of CK1 phosphorylation sites within Cx43 generally impairs mitochondrial oxygen consumption. We already showed a specific decrease of complex 1-mediated respiration in mitochondria isolated from inducible Cx43-knockout mice [7], which contain about 10% Cx43, and hypothesized that the mitochondrial amount of Cx43 is important for mitochondrial function. Interestingly, in the present study, we detected similar reductions of oxygen consumption in mitochondria isolated from Cx43^MAPKmut^ and Cx43^PKCmut^ mice containing either 73% or 9% Cx43, whereas the highest limitation in complex 1-mediated respiration was found in mitochondria from Cx43^CK1mut^ mice containing about 62% Cx43. Therefore, it is unlikely that the influence of Cx43 on mitochondrial respiration is caused by a reduction in the amount of the protein only; rather, the phosphorylation status of Cx43 is involved in the regulation of mitochondrial oxygen consumption. Since Cx43 phosphorylation at S262 is absent in Cx43^MAPKmut^ mice and the phosphorylation at this residue is increased in mitochondria of Cx43^PKCmut^ and Cx43^CK1mut^ mice, the importance of Cx43 S262 for respiration can presumably be neglected. The confinement of respiration correlated with the phosphorylation status at S325/328/330, which was higher in Cx43^MAPKmut^ than in Cx43^PKCmut^ and absent in Cx43^CK1mut^ mitochondria. The finding that the amount of complex 1 of the electron transport chain is enhanced in Cx43^CK1mut^ mice suggests that the efficiency of the electron transport chain is decreased in this mouse strain.

ROS formation is decreased after inhibition of Cx43 by carbenoxolone [68] or small interfering RNAs [77]. Mitochondrial Cx43 is involved in the regulation of MPTP (which occurs at the onset of reperfusion, leads to mitochondrial swelling, and finally cell death) as shown by an accelerated calcium-induced MPTP opening in cardiac mitochondria after inhibition of Cx43 with the mimetic peptide Gap27 or 18 alpha-glycyrrhetinic acid [8, 73]. In contrast, increased mitochondrial calcium retention capacity was measured in cardiac mitochondria upon Cx43 inhibition; however, these experiments were performed while blocking the mitochondrial calcium uniporter [20]. In the present study, ROS formation and calcium-induced MPTP opening were similar in WT, Cx43^MAPKmut^, Cx43^PKCmut^, and Cx43^CK1mut^ mitochondria. Taking into account that the mitochondrial amount of Cx43 significantly differs between the three mouse lines without having consequences for ROS formation and MPTP opening, the present study again emphasizes that not only the amount of Cx43 but also posttranslational modifications are important for mitochondrial function.

The role of Cx43 phosphorylation in cardiac *I/R* injury has been addressed in several studies. Whereas an alteration of the phosphorylation status of Cx43 is observed with ischemia, the specific phosphorylation of Cx43 on S325/328/330 [2, 37], S365 [2, 71], S368 [17, 76], and S373 [2, 14] depends on the model and the duration of ischemia. However, several studies demonstrate that ischemia is associated with dephosphorylation of Cx43, a lateralization of the protein from the gap junctions and a subsequent internalization of the protein [60]. As a consequence of *I*/*R*, the interactions between Cx43 and its binding partners are altered [44], an effect potentially contributing to decreased electrical and chemical gap junctional coupling during ischemia [46]. Yet, the present study did not directly investigate the effect of Cx43 phosphorylation on gap junctional coupling in myocardial *I*/*R* injury. An opening of Cx43-formed hemichannels is known to contribute to *I/R* injury [65, 81]. Hemichannel opening is regulated via Cx43 phosphorylation, e.g., the mutation of the PKC site S368 to alanine reduces hemichannel opening (for review see [52]) and astrocytes of Cx43^MAPKmut^ mice display diminished hemichannel activity [19]. Recently, it was shown that in a model of Duchenne muscular dystrophy the mutation of S325/328/330 to phospho-mimicking glutamic acids increased hemichannel opening [28]. However, the replacement of S325/328/330 to alanine or aspartate residues both enhances hemichannel opening [18]. Here, we describe decreased hemichannel activity in Cx43^PKCmut^ and Cx43^CK1mut^, but not in Cx43^MAPKmut^ cardiomyocytes. Therefore, we confirm previous data on the role of PKC-mediated Cx43 phosphorylation for hemichannel opening, whereas the contribution of MAPK and CK1 signaling on Cx43 hemichannel activity seems to be dependent on the model/cell type analyzed. It is interesting to note, that although probability of hemichannel opening was partially reduced, the extent of *I/R* injury was similar between the different mouse strains.

It is likely that Cx43 phosphorylation is involved in cardioprotection, since pharmacological strategies which reduce myocardial damage following *I*/*R* are often associated with preserved Cx43 phosphorylation [60]. Moreover, the mutation of S368 to alanine abolishes the cardioprotection by sphingosine-1-phosphate [48]. However, the similar infarct sizes following *I*/*R* we observed in all strains analyzed argue against an influence of Cx43 phosphorylation at MAPK, PKC, or CK1 target sites on myocardial *I*/*R* injury per se. Similar infarct sizes in WT and Cx43^CK1mut^ mice have already been demonstrated after myocardial *I*/*R* in animals less than 1 year old [70]. However, it must be considered that in the analyzed mouse strains—especially in Cx43^PKCmut^ mice—not only the phosphorylation of Cx43 but also the total amount of the protein is reduced. Previous studies in mice with 50% Cx43 demonstrate decreased myocardial infarction following permanent coronary ligation [36], but no influence of the reduced protein amount on infarct size after acute *I/R* [59, 62].

Data showing that ischemia induces dephosphorylation of Cx43 lead to the hypothesis that the cardioprotection by IPC may prevent such dephosphorylation, and indeed, IPC enhances Cx43 phosphorylation (S262 and S368) both in vitro and in vivo [56, 61, 72]. It is suggested that the cardioprotection by IPC is mediated at least in part via suppressing chemical gap junctional communication [46, 60], but also mitochondrial Cx43 seems to play a role [40, 55]. A reduction of Cx43 to about 50% in constitutive knockout mice is sufficient to abrogate IPC’s cardioprotection in vivo [62] and in vitro (present study with Cx43^Cre−ER(T)/fl^ mice); however, others demonstrate that IPC reduces infarct size in vitro in inducible Cx43 knockout mice expressing half the level of Cx43 [59]. In the present study, we showed that Cx43^PKCmut^ mice expressing around 40% of normal Cx43 levels were effectively protected by IPC in vitro, whereas in Cx43^CK1mut^ mice with 54%, the level of Cx43 of the cardioprotection by IPC was lost. These data argue that Cx43 expression levels alone cannot explain IPC effects and indicate the importance of posttranslational modifications. Our data indicate that the phosphorylation of Cx43 at MAPK and PKC-target sites is not essential for the cardioprotection by IPC, but highlight the role of CK1-targeted residues for the reduction of myocardial *I/R* damage. Moreover, the loss of infarct size reduction by IPC in Cx43^CK1mut^ mice correlated with the highest S262 phosphorylation of mitochondrial Cx43. Whereas opening of Cx43-formed hemichannels during ischemia is known to contribute to myocardial *I/R* damage, our finding of reduced hemichannel activity in Cx43^CK1mut^ mice makes it unlikely that hemichannel opening is involved in the loss of cardioprotection by IPC in these mice. Cardiomyocytes of Cx43^CK1mut^ mice—similar to Cx43^MAPKmut^ and Cx43^PKCmut^ mice—displayed reduced NCX currents, suggesting that targeting phosphorylation on the Cx43 C-terminal residues can control channel gating and affects the kinetic of the NCX, one of the regulators of intracellular calcium homeostasis. Ca^2+^ entry via reverse mode NCX contributes to myocardial *I/R* injury [31]. Since our data demonstrated reduced NCX currents in all three mouse lines with mutated Cx43 phosphorylation sites, NCX-mediated currents are unlikely to explain the loss of cardioprotection specifically in Cx43^CK1mut^ mice.

Mitochondria are important targets in the transduction of myocardial conditioning [6]. Reduction of mitochondrial Cx43 (without affecting gap junctional Cx43) abrogates pharmacological preconditioning [55], whereas the overexpression of mitochondrial Cx43 induces cytoprotection [43], pointing to an important role of the amount of mitochondrial Cx43 in cardioprotection. However, such direct correlation between the level of mitochondrial Cx43 and cardioprotection by IPC is not supported by the present data. Heterozygous Cx43-deficient mice have a specific deficit in ROS formation in response to diazoxide, and accordingly, the cardioprotection by pharmacological preconditioning with diazoxide is lost [24]. Yet, the present study demonstrates that neither the reduction of mitochondrial Cx43 by more than 50% nor the mutation of Cx43 phosphorylation sites modified ROS formation per se. In line with these findings, myocardial infarct size following *I*/*R* was similar among all mice strains. MPTP opening is regulated by Cx43, since pharmacological inhibition of Cx43 decreases the calcium retention capacity and thereby accelerates MPTP opening [8, 73]. In *I*/*R* injury, MPTP opening at reperfusion contributes to irreversible myocardial damage; a similar MPTP opening was found in Cx43^MAPKmut^, Cx43^PKCmut^, and Cx43^CK1mut^ mitochondria which went along with an unaltered infarct size following *I*/*R* but also argues against its importance for the attenuation of cardioprotection by IPC in Cx43^CK1mut^ mice. With reperfusion after prolonged ischemia, the combination of still elevated calcium concentrations and restored ATP levels via oxidative phosphorylation leads to cardiomyocyte hypercontracture and finally to cell death [21]. The decreased oxygen consumption measured in Cx43^CK1mut^ mitochondria might possibly limit the generation of ATP at reperfusion and may thereby be beneficial; however, similar infarct sizes in WT and Cx43^CK1mut^ hearts do not favor an involvement of CK1-mediated Cx43 phosphorylation in cardiomyocyte hypercontracture. Several studies show that in the cardioprotection by IPC preserved mitochondrial energetics by maintaining mitochondrial oxygen consumption limits cell death after myocardial *I*/*R* injury [6]. In the present study, impaired respiration was measured especially in Cx43^CK1mut^ mitochondria. Whereas this finding suggests that limited oxygen consumption in Cx43^CK1mut^ mitochondria may be important for the loss of infarct size reduction by IPC in Cx43^CK1mut^ mice in vitro, the exact contribution of mitochondrial Cx43 (total amount and phosphorylated Cx43) towards the cardioprotection by IPC remains unclear.

Study limitations—1. In the present study, we used genetically modified mice in which Cx43 phosphorylation sites are constitutively altered. These mice may carry some unrecognized additional mutations, which may also influence cellular and/or mitochondrial function.

2. Myocardial infarction after *I*/*R* injury may differ between isolated buffer-perfused hearts—as performed in the present study—and the in vivo situation. Therefore, we cannot exclude that Cx43^MAPKmut^, Cx43^PKCmut^, and Cx43^CK1mut^ mice will show a different response towards a preconditioning stimulus in the in vivo situation. Thus, the evaluation of infarct size without and with IPC in vivo will represent an important extension of the present study.

3. In addition, it has to be noted that mitochondrial function and electrophysiological measurements were performed at 25 °C or room temperature. Whereas mitochondrial oxygen consumption is generally lower at 25 °C compared to 37°, the temperature sensitivity of mitochondrial respiration is dependent on the type of substrates and is lowest for glutamate and malate [38], the complex 1 substrates used in the present study. While data show that Cx26-formed hemichannels are sensitive to changes in the temperature [74, 80], Cx43 hemichannel gating mechanisms are not affected by temperature [51, 57], and therefore, the majority of studies investigating Cx43 hemichannels were commonly performed at room temperature [3, 33, 35, 45, 53, 60]. Importantly, since experiments on mitochondrial function and electrophysiological recordings were carried out at the same temperatures, differences in respiration and hemichannel activity between the mouse strains cannot be attributed to temperature variances.

**Fig. 6:**
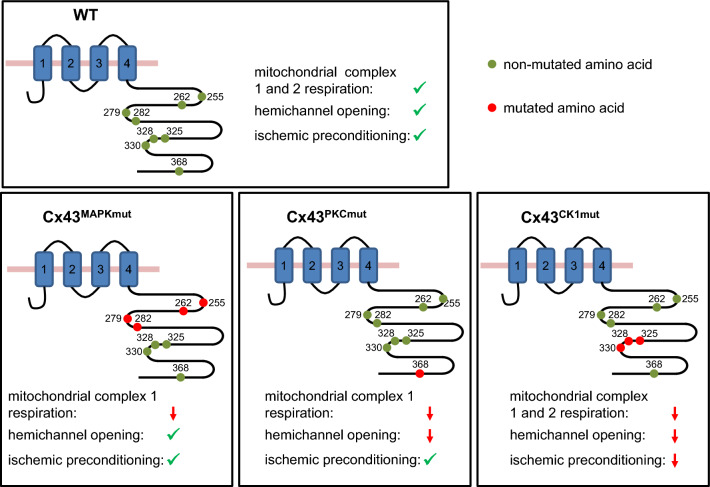
The role of Cx43 phosphorylation for specific cellular functions. The scheme shows the topology of Cx43 including the transmembrane domains (1–4) and important phosphorylation sites, which are indicated by filled circles and are numbered according to their positions. ↓: decreased/impaired function, ✓: normal function

Taken together, our data show that the mutation of Cx43 phosphorylation sites targeted by MAPK, PKC, or CK1 to non-phosphorylatable residues affects the amount and the phosphorylation of Cx43 at amino acids not directly targeted by the mutations and thereby emphasizes the complex interplay between posttranslational Cx43 modifications and the amount of the Cx43 protein. The alterations of the amounts/phosphorylation of Cx43 have consequences for specific cellular function, since mutation of Cx43 phosphorylation sites for MAPK, PKC, and CK1 sites reduces mitochondrial respiration, mutation of PKC and CK1 sites decreases hemichannel activity, and the mutation of Cx43 residues targeted by CK1 abrogates the infarct size reduction by IPC (Fig. 6). Therefore, in addition to Cx43 phosphorylation at S262 and S368, phosphorylation at S325/328/330 is involved in mediating IPC’s cardioprotection, an effect independent from the protein amount of Cx43 and Cx43-mediated hemichannel opening.

## Supplementary Information

Below is the link to the electronic supplementary material.

Supplementary file1 (PDF 1082 kb)

Supplementary file2 (PDF 335 kb)

## Data Availability

Not applicable.

## Abbreviations

AKT: Protein kinase B
CamKII: Ca^2^^+^/calmodulin kinase II
CK1: Casein kinase 1
Cx43: Connexin 43
CsA: Cyclosporine A
EDP: End-diastolic pressure
ERK: Extracellular-signal related kinase
GAPDH: Glyceraldehyde 3-phosphate dehydrogenase
IFM: Interfibrillar mitochondria
IPC: Ischemic preconditioning
I/R: Ischemia/reperfusion
LV: Left ventricle
LVDP: Left-ventricular developed pressure
NCX: Sodium/calcium exchanger
MAPK: Mitogen-activated protein kinase
MnSOD: Manganese superoxide dismutase
MPTP: Mitochondrial permeability transition pore
p34cdc2: P34^cdc2^/cyclin B kinase
PKA: Protein kinase A
PKC: Protein kinase C
ROS: Reactive oxygen species
S: Serine
SSM: Subsarcolemmal mitochondria
Src: Pp60src kinase
TTC: Triphenyltetrazolium chloride
WT: Wild type
ZO-1: Zonula occludens 1
