# Supplementary material for: Connexin 43 phosphorylation by casein kinase 1 is essential for the cardioprotection by ischemic preconditioning
Source: Basic Res Cardiol. 2021 Mar 22;116(1):21. doi: 10.1007/s00395-021-00861-z (PMC7985055; doi:10.1007/s00395-021-00861-z)
Supplement: Supplementary file 1 — Supplementary file1 (PDF 1082 kb) [file 395_2021_861_MOESM1_ESM.pdf]

Supplementary online material

Connexin 43 phosphorylation by casein kinase 1 is essential for the cardioprotection by ischemic preconditioning

Christine Hirschhäuser, Alessio Lissoni[#], Philipp Maximilian Görge, Paul D. Lampe PhD*, Jacqueline Heger PhD, Klaus-Dieter Schlüter PhD, Luc Leybaert PhD[#], Rainer Schulz MD, Kerstin Boengler PhD[**]

Institute of Physiology, Justus-Liebig University, Giessen, Germany
[#] Department of Basic Medical Sciences - Physiology group, Faculty of Medicine and Health Sciences, Ghent University, Ghent, Belgium
* Fred Hutchinson Cancer Research Center, Seattle, Washington, US

Kerstin.boengler@physiologie.med.uni-giessen.de

Material and Methods

Mitochondria isolation

Subsarcolemmal mitochondria were isolated from left ventricular tissue of WT, $Cx43^{MAPKmut}$, $Cx43^{PKCmut}$ and $Cx43^{CK1mut}$ mice. Mice were anaesthetized with 5% isoflurane and killed by cervical dislocation. Hearts were quickly excised; the left ventricles were separated and were minced in 5 ml buffer A (in mol/L: KCl 100, 3-[N-Morpholino]-propanesulfonic acid (MOPS) 50, MgSO4 5, ATP 1, EGTA 1, pH 7.4) + 4% bovine serum albumine followed by homogenization with a Potter-Elvehjem tissue homogenizer. The homogenate was centrifuged for 10 min at 800 g. The resulting supernatant, which contained the SSM, was centrifuged for 10 min at 8000 g. The sedimented mitochondria were washed in buffer A and resuspended in a small volume of homogenation buffer, which is buffer A without ATP. Interfibrillar mitochondria (IFM), which contain only a small amount of Cx43, were also isolated by resuspending the sediment of the first centrifugation in buffer A (10 ml/g tissue). Eight U/g of the protease nagarse were added and incubated at 4°C for 1 min. After disruption with a Potter-Elvejhem tissue homogenizer and centrifugation for 10 min at 800 g the supernatant was centrifuged for 10 min at 8000 g to collect the IFM. The resulting mitochondria were washed by resuspension in buffer A, they were centrifuged at 8000 g for 10 min, and finally resuspended in buffer A. All steps were performed at 4°C.

These SSM preparations were used to study respiration, ROS formation and calcium-induced MPTP opening. To analyse the amount and phosphorylation of Cx43 by Western blot, SSM and IFM were further

purified by layering them on top of a 30% Percoll solution in isolation buffer (in mmol/L: sucrose 250; HEPES 10; EGTA 1; pH 7.4) and subsequent ultracentrifugation at 35.000 g for 30 min at 4°C. The lower mitochondrial band was collected, washed twice in isolation buffer by centrifugation at 8000 g for 5 min, and the purified mitochondria were stored at -80°C.

Western Blot analysis

Left ventricular (LV) or right ventricular (RV) tissue samples and SSM or IFM purified by Percoll gradient ultracentrifugation were lysed in 1X NP40 buffer (25 mmol/L Tris, 150 mmol/L NaCl, 1 mmol/L EDTA, 1% NP-40, 5% glycerol, pH 7.4) supplemented with 1X PhosStop and Complete inhibitors (Roche, Basel, Switzerland) as well as 1 µM neocuproine. Protein concentration was determined using the Lowry assay. Forty µg LV proteins or mitochondrial proteins were electrophoretically separated on 10% Bis-Tris gels and proteins were transferred to nitrocellulose membranes. For the detection complexes of the electron transport chain, only 10 µg protein were run on the gel and samples were denatured at 37°C for 10 min instead of 5 min 95°C used for all other samples. Protein extracts from WT and mutated mice were always run on one gel. When needed, membranes were cut horizontally in order to detect 2 or more proteins on the same membrane. This procedure helps 1) to minimize the amount of proteins needed for the analysis (an important aspect considering the low amount of mitochondrial proteins, which can be isolated from mouse left ventricular tissue. Therefore, cutting the membranes helps to decrease the number of animals needed for the overall study and thereby are in agreement with the 3R principle which is a request by the university and government) and 2) improves the quantification of the data, since inaccuracies due to pipetting errors occurring when the protein of interest and the housekeeping protein are derived from separate gels are prevented. After the detection of phosphorylated Cx43, membranes were reprobed with antibodies against total Cx43 and with antibodies against GAPDH after the detection of total Cx43.

After blocking, membranes were incubated with antibodies against phosphorylated Cx43 (rabbit polyclonal anti-Cx3043-S262 (Santa Cruz, Santa Cruz, CA), dilution 1:2000; rabbit polyclonal anti-Cx43-S368 (Cell Signaling, Danvers, MA), dilution 1:250; rabbit polyclonal anti-Cx43-S325/328/330 (Fred Hutchinson Cancer Research Center, Seattle, WA), dilution 1:2000; rabbit polyclonal anti-Cx43-S365 (Fred Hutchinson Cancer Research Center), dilution 1:250; rabbit polyclonal anti-Cx43-S373 (Fred Hutchinson Cancer Research Center), dilution 1:250), total Cx43 (rabbit polyclonal anti-human/rat Cx43 antibodies (Sigma, Munich, Germany), dilution 1:1000 or the OxPhos Rodent WB Antibody Cocktail (Invitrogen, dilution 1:2000). Total Cx43 was normalized to the protein contents of the glyceraldehyde-3-phosphate dehydrogenase (mouse monoclonal anti-GAPDH, HyTest, Turku, Finland, dilution 1:30000) in LV tissue samples and to the manganese superoxide dismutase (rabbit polyclonal anti-human manganese superoxide dismutase antibodies (Merck Millipore, Darmstadt, Germany), dilution 1:1000) in mitochondrial samples. After washing and incubation with the respective secondary antibodies, immunoreactive signals were detected by chemiluminescence (SuperSignal West Pico or SuperSignal West Femto Chemiluminescent

Substrate, ThermoFisher) and were quantified using Scion Image software (Frederick, MD). Antibodies were re-used several times, therefore, different exposition times of the membranes were required and accordingly, the quantification resulted in different arbitrary units for the same phosphorylation site(s) in different strains. Therefore, only relative alterations not the absolute immunoreactivity values can be compared. Mitochondrial proteins were routinely screened for the presence of proteins derived from other cell compartments (Na^+/K^+-ATPase for the sarcolemma, GAPDH for the cytosol and HDAC2 (histone deacytelase 2) for the nucleus) and only mitochondrial proteins which were not contaminated by these marker proteins were used for the analysis of Cx43 phosphorylation (supplementary Fig. 3).

Mitochondrial oxygen consumption

Oxygen consumption was measured as described previously (Boengler, BJP2017). One hundred µg/ml SSM were transferred to incubation buffer (containing in mmol/L: 125 KCl, 10 Tris (titrated with MOPS), 1.2 Pi (titrated with Tris), 1.2 $MgCl_2$, 0.02 EGTA (titrated with Tris), pH 7.4). Complex 1-mediated respiration was analyzed with a Clark-type oxygen electrode (Oxygen meter 782, Strathkelvin, Glasgow, UK) at 25 °C in incubation buffer supplemented with 5 mmol/L glutamate and 2.5 mmol/L malate, whereas complex 2-mediated respiration was measured in the presence of 5 mmol/L succinate and 2µmol/L rotenone (to inhibit complex 1-mediated respiration). After recording the basal oxygen consumption, respiration was stimulated by the addition of 40 µmol/L ADP. Subsequently, 0.27mol/L N,N,N′,N′-tetramethyl-p-phenylenediamine (TMPD) and 0.82 mmol/L ascorbate were added, followed by the administration of 100 nmol/L carbonyl cyanide 4-(trifluoromethoxy)phenylhydrazone (FCCP). Oxygen consumption was analyzed in nmol O_2*min^{-1}*mg $protein^{-1}$. In each experiment SSM isolated from WT mice and mice with mutated Cx43 phosphorylation sites were analyzed.

ROS formation

The fluorescence of 50 µg SSM in incubation buffer supplemented with 5 mmol/L glutamate and 2.5 mmol/L malate, 50 µmol/L Amplex UltraRed (Invitrogen, Eugene, OR), and 0.1 U/ml horseradish peroxidase was measured continuously for 4 min with a Cary Eclipse spectrophotometer (Agilent Technologies, Santa Clara, CA) at the excitation/emission wavelengths of 565/581 nm, respectively. Background fluorescence of the buffer without mitochondria was subtracted and the slope (fluorescence in arbitrary units/time (4 min)) was calculated. In each experiment SSM isolated from WT mice or mice with mutated Cx43 phosphorylation sites were analyzed.

Calcium-induced opening of the mitochondrial permeability transition pore

Extramitochondrial calcium of 100 µg/ml SSM was analyzed at 25°C in incubation buffer with glutamate/malate as substrates for complex 1, 5 µmol/L EGTA, 40 µmol/L ADP, 0.5 µmol/L Calcium Green 5N (Invitrogen, Carlsbad, CA) with a Cary Eclipse spectrophotometer at excitation and emission wavelengths of 500 and 530 nm, respectively. Five µmol/L $CaCl_2$ were added every third minute until a sudden increase in Calcium Green 5 N fluorescence occurred reflecting MPTP opening. As a positive control, measurements were performed in the presence of 1µmol/L Cyclosporin A (CsA), which inhibits MPTP opening. In each experiment SSM isolated from WT mice or mice with mutated Cx43 phosphorylation sites were analyzed.

Electrophysiological recordings

Adult C57BL6 (WT), $Cx43^{MAPKmut}$, $Cx43^{PKCmut}$ and $Cx43^{CK1mut}$ mice, were heparinized (5000 IU/kg IP) and sacrificed by cervical dislocation. Following thoracotomy, the heart was quickly excised and then transferred to a Langendorff apparatus and perfused at a constant flow (~3 ml/min) and temperature (37°C). Left ventricular isolated cardiomyocytes were isolated as previously described (Lissoni et al., 2019). Ca^{2+}-tolerant cells were used for the experiment for the next 6 hours.
Isolated ventricular cardiomyocytes were studied under whole-cell voltage-clamp to record membrane currents. Cells were kept in standard Tyrode solution (in mmol/L): 137 NaCl, 5.4 KCl, 1 $CaCl_2$, 0.5 $MgCl_2$, 10 glucose, 11.8 HEPES and pH adjusted to 7.4). Unitary current events were triggered by applying 10 mmol/L caffeine to the extracellular solution. The standard pipette solution for whole cell recording was composed of (in mmol/L): 120 K-aspartate, 10 KCl, 10 NaCl, 10 Hepes, 0.5 $MgCl_2$, 5 MgATP and pH adjusted to 7.2.

Single channel recordings were performed with an EPC 7 PLUS patch-clamp amplifier (HEKA Elektronik, Lambrecht/Pfalz, Germany). Data were acquired at 4 kHz using a NI USB-6221 data acquisition device from National Instruments (Austin, TX, USA) and WinWCP or WinFluor software (credited to Dr. J. Dempster, Strathclyde Institute). All currents in whole cell configuration were filtered at 1 kHz (7-pole Besselfilter). Membrane potentials were corrected for the liquid junction potentials when present. Patch pipette resistance was 2 – 3 MΩ.
For single channel analysis, macroscopic and holding currents were subtracted from the recorded current traces making use of a novel cross-correlation and threshold detection algorithm software. Open probability was calculated as ∑ *(channel open durations)/analysis time window*. Sarcoplasmic reticulum Ca^{2+} content was measured by integrating the inward NCX current during fast caffeine application (10 mM).

Isolation of ventricular cardiomyocytes

Ventricular cardiomyocytes were isolated from wildtype mice as previously described [2]. Mice were anesthetized with 4-5% isoflurane, hearts were excised and immediately mounted on the cannula of a Langendorff perfusion system. Hearts were perfused at 37°C with 5ml of a buffer containing in mM NaCl 110, KH_2PO_4 1.2, KCl 2.6, $MgSO_4$ 1.2, HEPES 25, glucose 11,pH 7 .4. Subsequently, perfusion was continued for 28 min with recirculation with 50 ml of the perfusate supplemented with 25 mg collagenase (Worthington, Lakewook, NJ) and 25 µM $CaCl_2$ and a velocity of 1 drop/s. Ventricular tissue was minced and incubated for another 5 min with 5 ml of the perfusate. The resulting suspension was filtered through a nylon mesh (200 µm) and was washed four times by centrifugation (1min, 16g). Cells were resuspended in the perfusate, in which the $CaCl_2$ concentration was increased to 125 µM, 250 µM, 500 µM and 1000 µM and were then plated on laminin-coated (5µg/ml) cell culture dishes for 1h. After that, cell cultures were washed to remove round and non-attached cells. Subsequently, cells were cultivated for 2h and 6h in cultivation medium (in mM NaCl 110, KCl 2.6, KH_2PO_4 1.2, $MgSO_4$ 1.2, HEPES 25, glucose 11, $CaCl_2$ 1.5 supplemented with 1% fetal calf serum and 2% penicillin/streptomycin, pH 7.4, gased with 5% CO2) to which 30 µM CKI-7 (Tocris, UK, CK1 inhibitor, [1]), 100 nM GF 109203X (Abcam, PKC inhibitor [3]) or the respective amounts of DMSO as control were added. After the incubations, cells were washed with phosphate-buffered saline and proteins were extracted by adding 100 µl 2X cell lysis buffer (50 mM Tris, 300 mM NaCl, 2 mM EDTA, 2% NP-40, 10% glycerol, pH 7.4) and benzonase (dilution 1:1000). Twenty µg proteins were electrophoretically separated and characterized by Western blot analysis as described above.

Ischemia/reperfusion in vitro

WT, $Cx43^{MAPKmut}$, $Cx43^{PKCmut}$, $Cx43^{CK1mut}$ and $Cx43^{Cre-ER(T)/fl}$ mice were anaesthetized with 5% isoflurane and killed by cervical dislocation. Hearts were quickly excised and the aorta was cannulated for retrograde perfusion with an Aortic Cannula for mouse hearts (Ø 1 mm, Hugo Sachs Elektronik- Harvard Apparatus, March, Germany) connected to a Langendorff perfusion system. Hearts were perfused with 37°C warm modified Krebs Henseleit buffer (containing in mmol/L: NaCl 118, KCl 4.7, $MgSO_4$ 0.8, KH_2PO_4 1.2, glucose 5, $CaCl_2$ 2.5, $NaHCO_3$ 25, pyruvate 1.9, continuously gassed with 95% O_2, 5% CO_2, pH 7.4) at a constant perfusion pressure of 70 mmHg (transduced by a Replacement Transducer Head for APT300 Pressure Transducer, Hugo Sachs Elektronik- Harvard Apparatus). A balloon was inserted into the left ventricle and was connected to a pressure transducer (Combitrans 1-fach Set Mod.II University Giessen, B. Braun, Melsungen, Germany) for assessment of ventricular performance. The balloon was inflated to yield a left ventricular end-diastolic pressure of 12-14 mmHg, which was kept constant thereafter. Hearts were paced during measurements at 600 bpm. Enddiastolic and left ventricular developed pressure (LVDP, systolic pressure – diastolic pressure) were recorded. Perfused hearts underwent a stabilization period of 5 min. Ischemia was induced by stopping flow and pacing. Hearts underwent 45 min ischemia followed by 2 h reperfusion. The IPC protocol is comprised of three cycles of 3 min ischemia, 5 min reperfusion followed

by 45 min ischemia and 2 h reperfusion. At the end of reperfusion, hearts were removed from the perfusion apparatus and were frozen at -20°C for 30 min. Hearts were cut in 7-8 slices, incubated in 1.2 % triphenyl-tetrazolium chloride for 20 min at 37°C and were then fixated in 7% formalin at room temperature overnight. Digital images were taken from both sides of the heart slices with a M60 microscope (Leica, Wetzlar, Germany) at 2.5 fold magnification. Infarct size was determined by planimetrie using the Leica Application Suite LAS version 4.6 (Leica).

Supplementary Figures

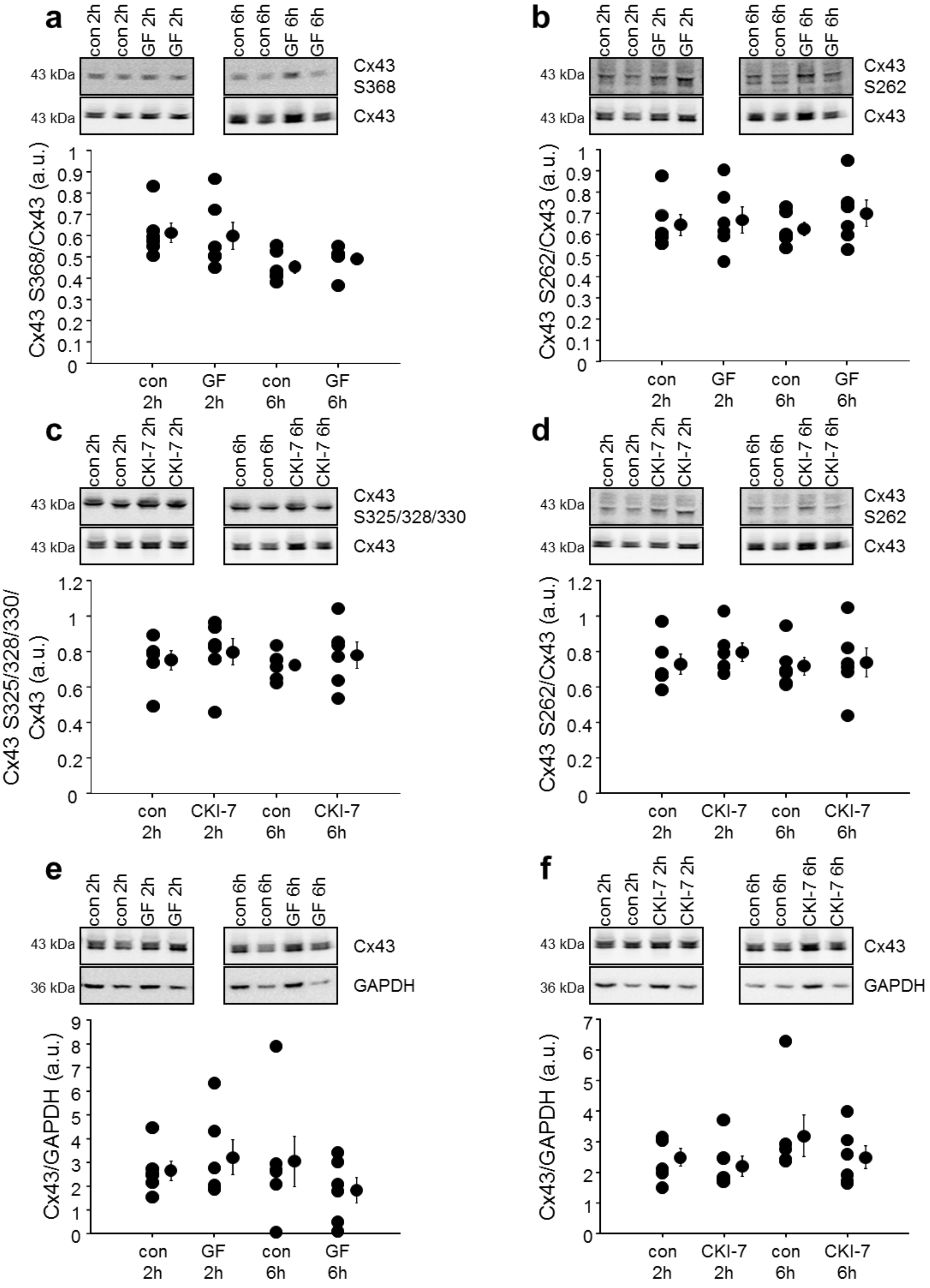

Supplementary figure 1 Effect of PKC or CK1 inhibition on the expression and phosphorylation of Cx43
Left ventricular cardiomyocytes were isolated from wildtype mice and were incubated with inhibitors of PKC (GF 109203X (GF), 0.1 µM) or CK1 (CKI-7, 30 µM) or under control conditions for 2h and 6h. Expression and phosphorylation of Cx43 at serine 368 (S368, a), serine 262 (S262, b, d), serines 325/328/330 (S325/328/330, c) were analyzed by Western blot. Original Western blot data for phosphorylated and total Cx43 as well GAPDH (glyceraldehyde-3-phosphate dehydrogenase, used for normalization) are presented. Ratios of phosphorylated Cx43 over total Cx43 (a-d), or total Cx43 over GAPDH (e-f) are shown in arbitrary units (a.u.) as individual data points and as mean values ± SEM, n=6, p=ns, paired t-test.

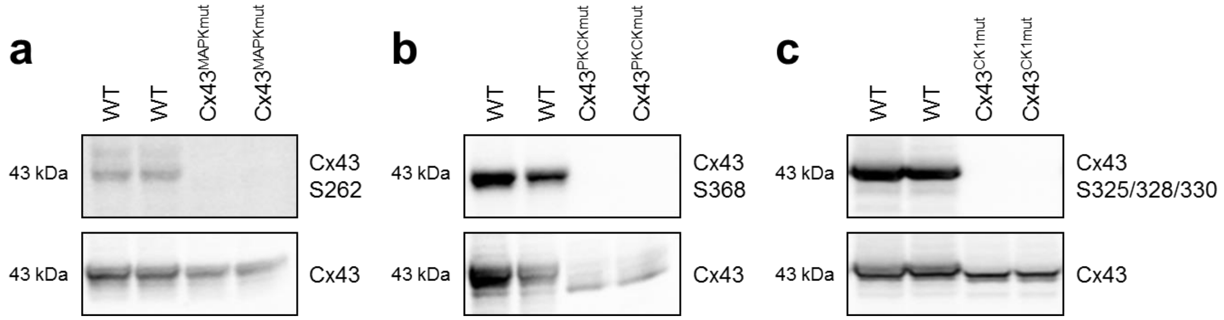

Supplementary figure 2 Specificity of the antibodies directed against phosphorylated Cx43
Western Blot analysis was performed for Cx43 phosphorylated at S262 in left ventricular (LV) total proteins extracted from Cx43^{MAPKmut} mice (**a**), for Cx43 phosphorylated at S368 in LV total proteins extracted from Cx43^{PKCmut} mice (**b**), for Cx43 phosphorylated at S325/328/330 in LV total proteins extracted from Cx43^{CK1mut} mice (**c**) and for total Cx43 in protein extracts of all genotypes. Wildtype (WT) proteins were used as controls. WT: n=6; Cx43^{MAPKmut}: n=6; Cx43^{PKCmut}: n=6; Cx43^{CK1mut}: n=6.

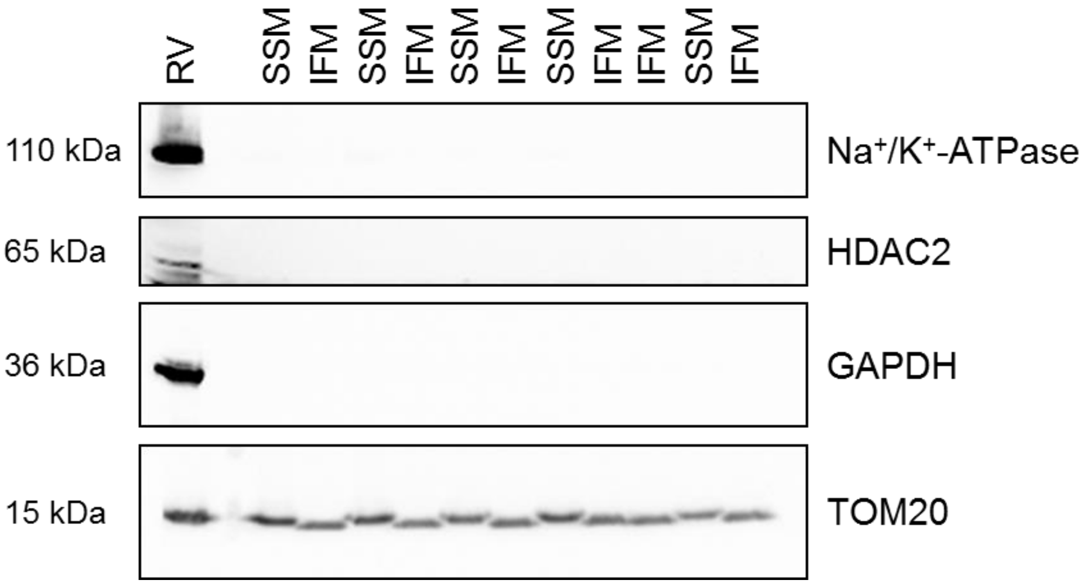

Supplementary figure 3 Purity of isolated mitochondrial proteins

Subsarcolemmal (SSM) and interfibrillar mitochondria (IFM) were isolated from the left ventricles of $Cx43^{CK1mut}$ mice. Western blot analysis was performed for Na^+/K^+-ATPase (marker protein of the sarcolemma), HDAC2 (histone deacytelase 2, nucleus) and GAPDH (glyceraldehyde-3-phosphate dehydrogenase, cytosol) and TOM20 (translocase of the outer membrane 20, mitochondria). A right ventricular total protein extract (RV) was used as a positive control. The purity of the isolated mitochondria is demonstrated by the absence of Na^+/K^+-ATPase, HDAC2, GAPDH and presence of TOM20 immunoreactivities in the SSM and IFM preparations. The purity of all mitochondrial preparations as shown here exemplary for $Cx43^{CK1mut}$ mitochondria was controlled prior to the analysis of the amount and phosphorylation of Cx43.

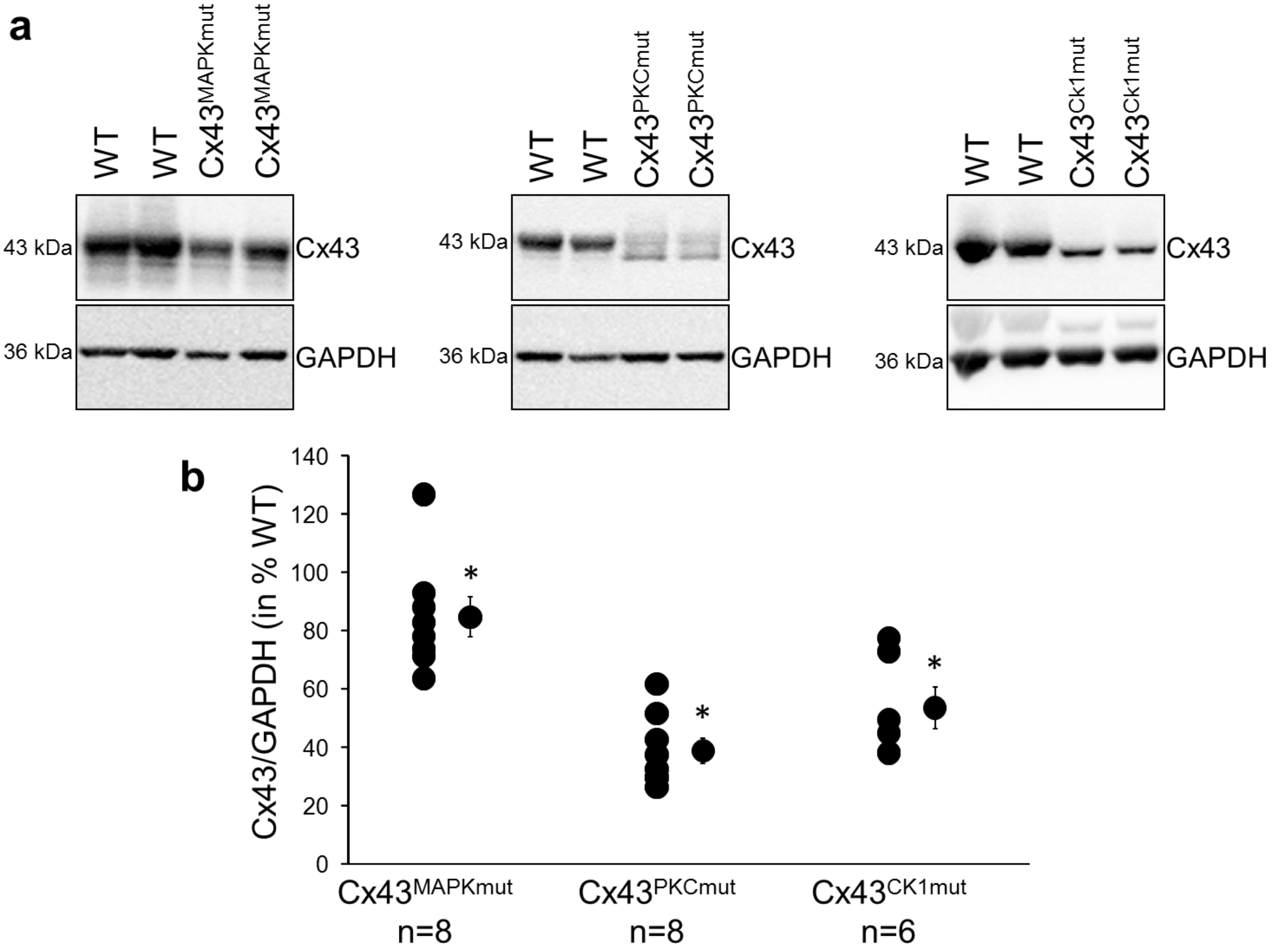

Supplementary figure 4 Amount of Cx43 in LV proteins in Cx43^{MAPKmut}, Cx43^{PKCmut} and Cx43^{CK1mut} mice

a: Total proteins were isolated from left ventricular (LV) tissue of wildtype (WT), Cx43^{MAPKmut}, Cx43^{PKCmut} and Cx43^{CK1mut} mice and Western Blot analysis was performed for total Cx43 and the marker protein GAPDH (glyceraldehyde-3-phosphate dehydrogenase).

b: The ratio of Cx43 over GAPDH is shown in % of that in WT mice. The number of WT mice was n=8 for Cx43^{MAPKmut}, n=8 for Cx43^{PKCmut} and n=6 for Cx43^{CK1mut} mice. *: $p<0.05$ vs. WT, non-parametric rank-sum test.

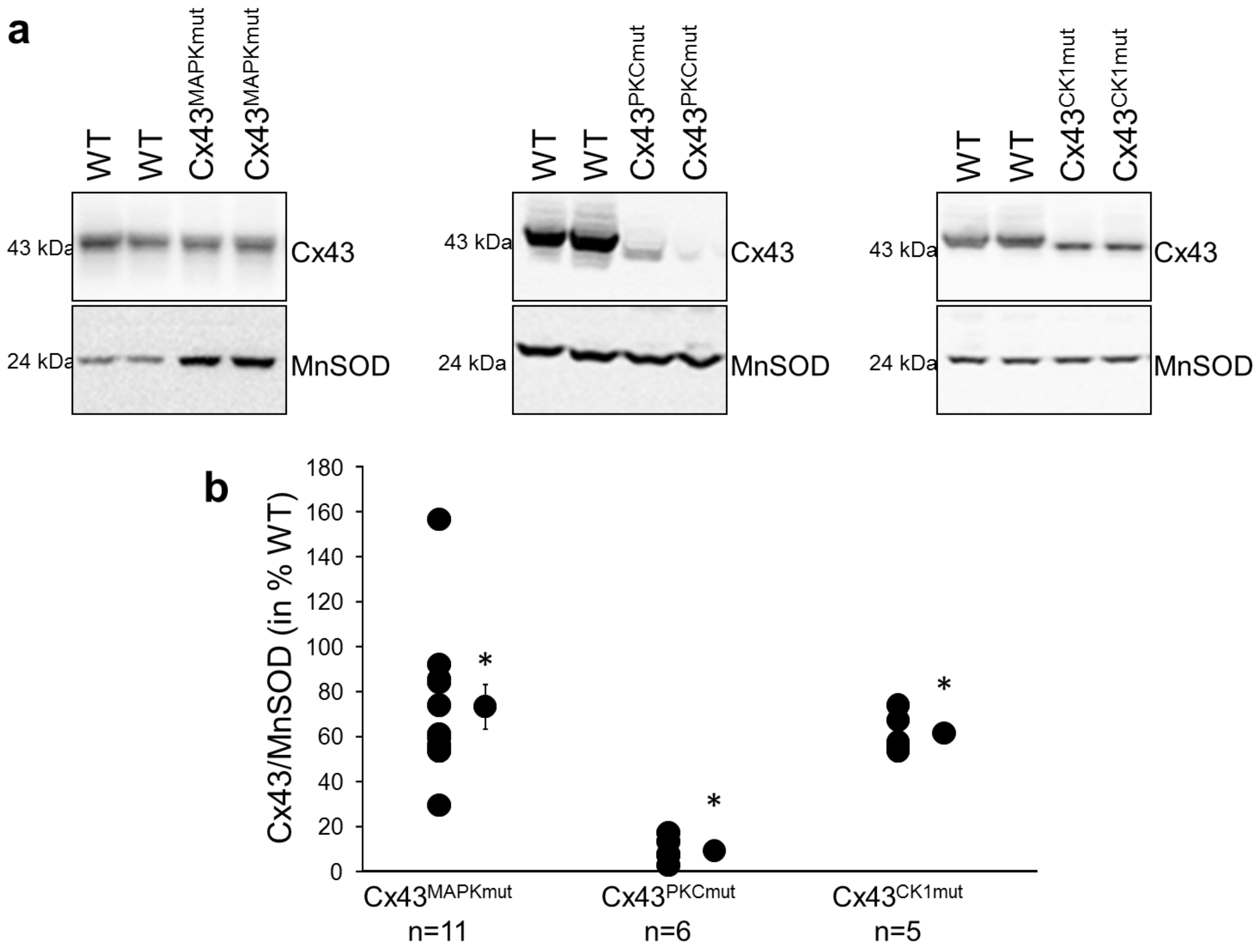

Supplementary figure 5 Mitochondrial amount of Cx43 in $Cx43^{MAPKmut}$, $Cx43^{PKCmut}$ and $Cx43^{CK1mut}$ mice
a: SSM were isolated from left ventricular (LV) tissue of wildtype (WT), $Cx43^{MAPKmut}$, $Cx43^{PKCmut}$ and $Cx43^{CK1mut}$ mice and Western Blot analysis was performed for total Cx43 and the mitochondrial marker protein MnSOD (manganese superoxide dismutase).
b: The ratio of Cx43 over MnSOD is shown in % of that in WT mice. The number of WT mice was n=8 for $Cx43^{MAPKmut}$, n=6 for $Cx43^{PKCmut}$ and n=6 for $Cx43^{CK1mut}$ mice. *: $p<0.05$ vs. WT, non-parametric rank-sum test.

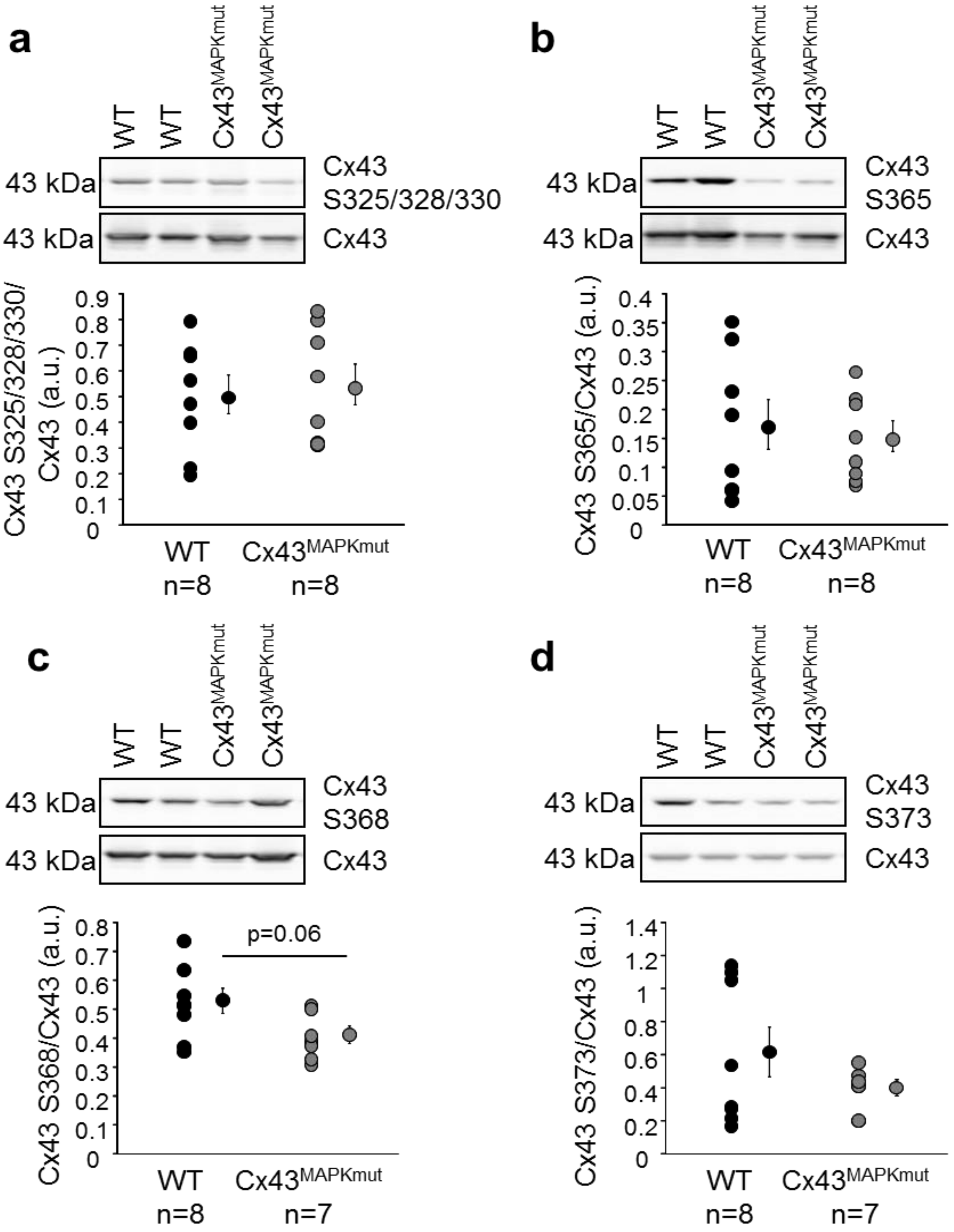

Supplementary figure 6 Phosphorylation of Cx43 in LV total protein samples in WT and Cx43^{MAPKmut} mice

Western Blot analysis was performed for Cx43 phosphorylated at S325/328/330 **(a)**, S365 **(b)**, S368 **(c)**, and S373 **(d)** as well as total Cx43 on proteins extracted from left ventricular (LV) tissue of wildtype (WT) and Cx43^{MAPKmut} mice. Both original Western Blot images (upper panels) and data in which the ratio of phosphorylated over total Cx43 is shown (lower panels, both individual data points and mean values ± SEM) are presented (unpaired t-test).

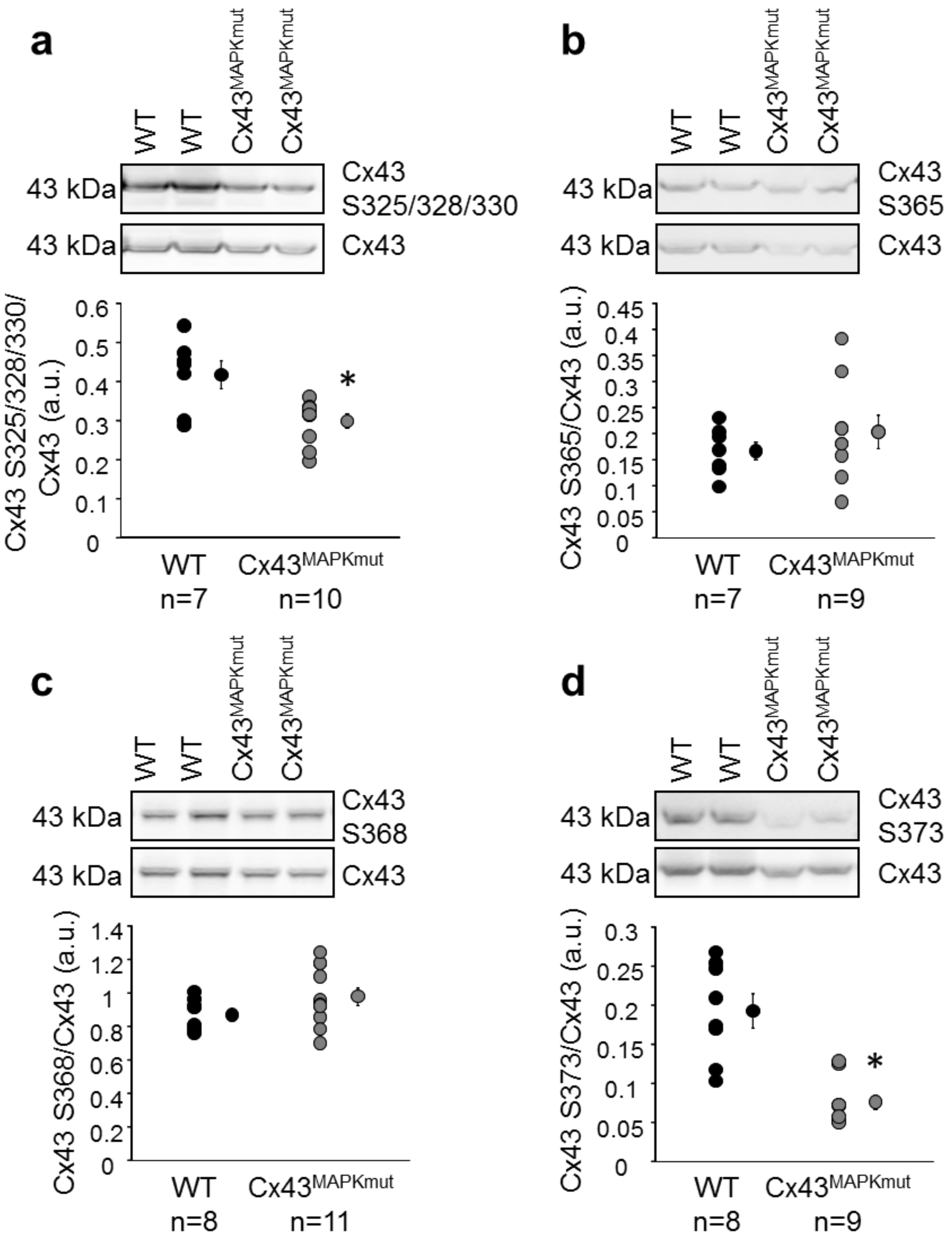

Supplementary figure 7 Cx43 phosphorylation in SSM in WT and Cx43^{MAPKmut} mice

Western Blot analysis was performed for Cx43 phosphorylated at S325/328/330 (**a**), S365 (**b**), S368 (**c**), and S373 (**d**) as well as total Cx43 on proteins extracted from left ventricular (LV) SSM of wildtype (WT) and Cx43^{MAPKmut} mice. Both original Western Blot images (upper panels) and data in which the ratio of phosphorylated over total Cx43 is shown (lower panels, both individual data points and mean values ± SEM)) are presented,* $p<0.05$ (unpaired t-test).

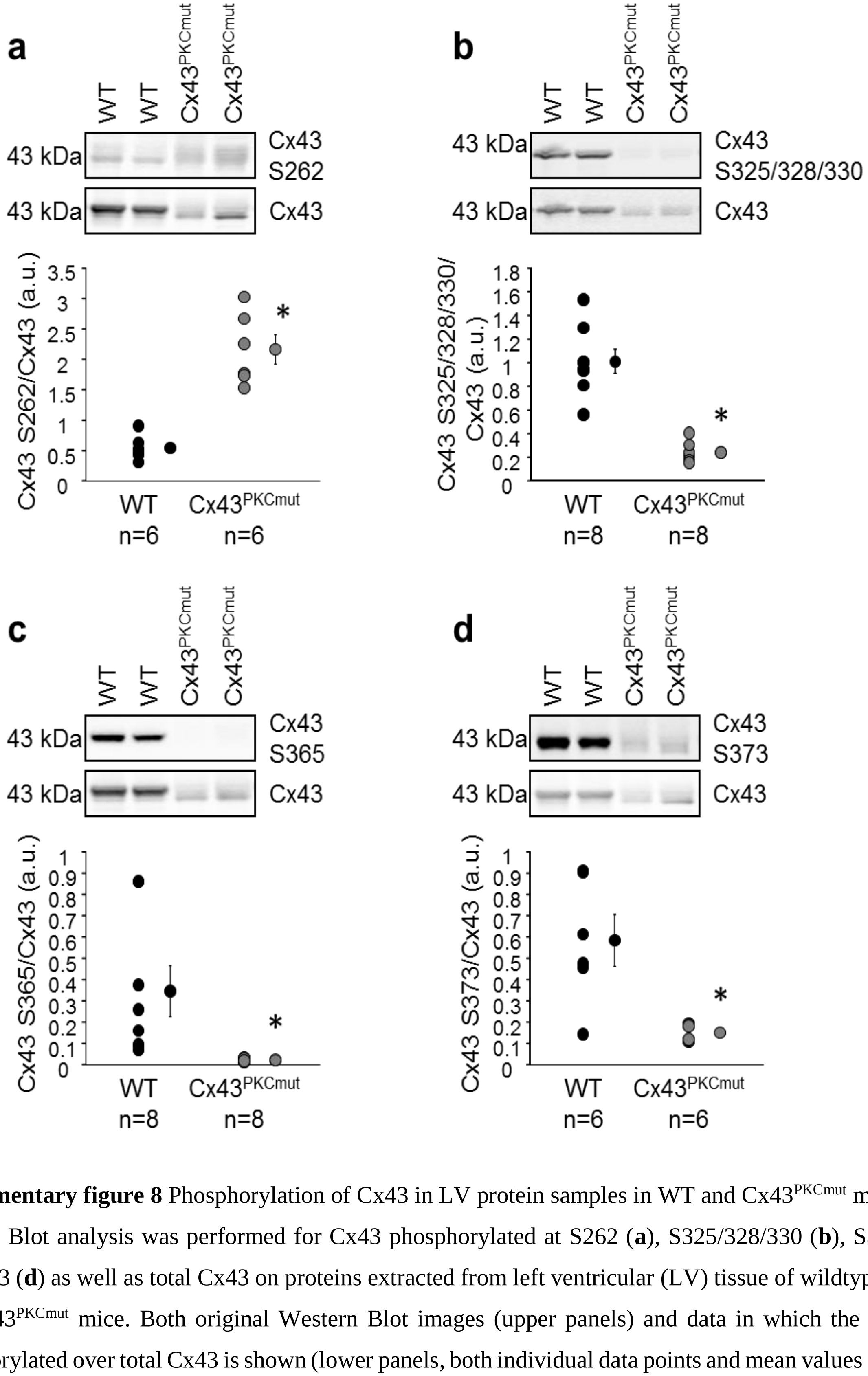

Supplementary figure 8 Phosphorylation of Cx43 in LV protein samples in WT and $Cx43^{PKCmut}$ mice
Western Blot analysis was performed for Cx43 phosphorylated at S262 (**a**), S325/328/330 (**b**), S365 (**c**), and S373 (**d**) as well as total Cx43 on proteins extracted from left ventricular (LV) tissue of wildtype (WT) and $Cx43^{PKCmut}$ mice. Both original Western Blot images (upper panels) and data in which the ratio of phosphorylated over total Cx43 is shown (lower panels, both individual data points and mean values ± SEM) are presented. *: p<0.05 (unpaired t-test).

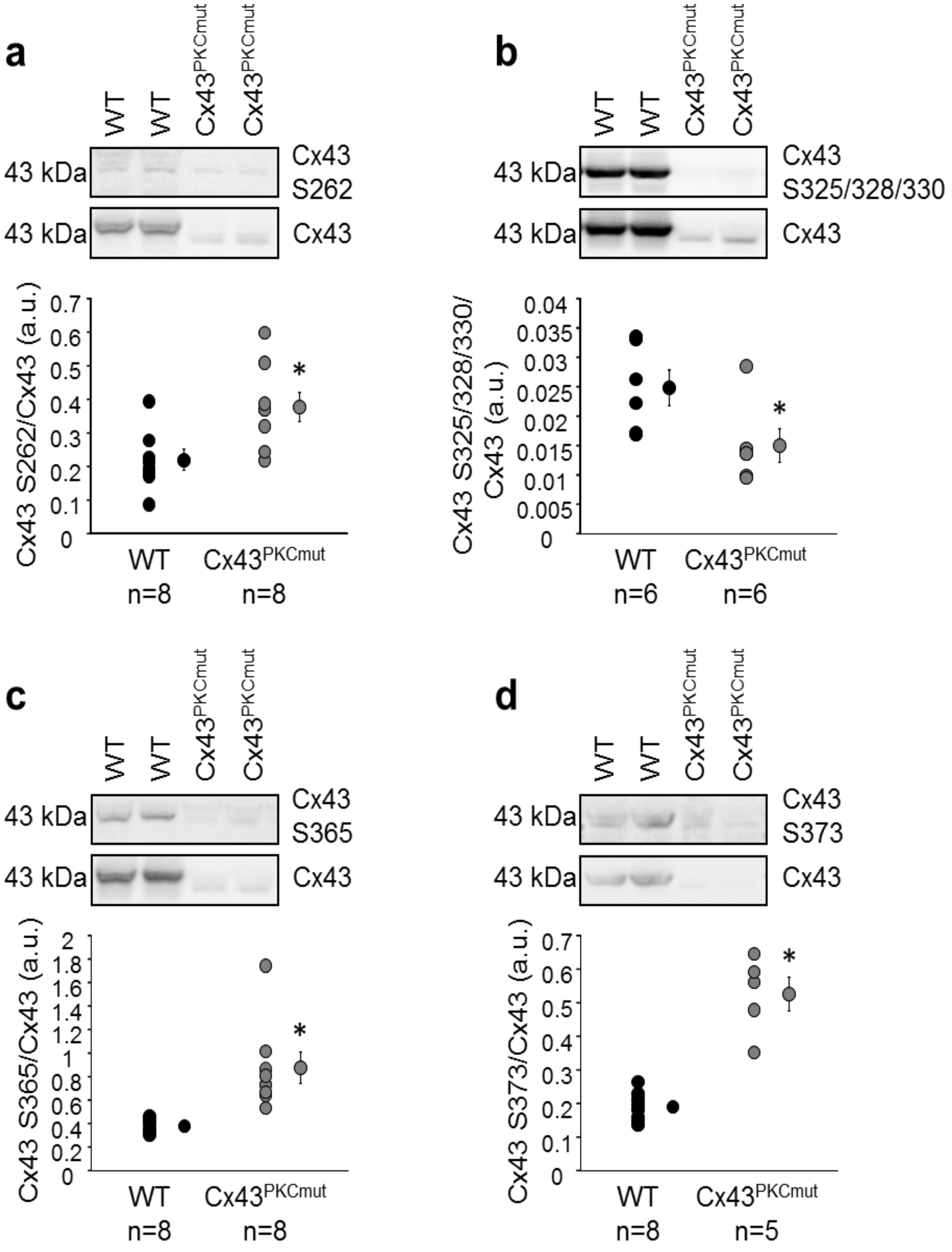

Supplementary figure 9 Phosphorylation of Cx43 in SSM in WT and Cx43^{PKCmut} mice

Western Blot analysis was performed for Cx43 phosphorylated at S262 (**a**), S325/328/330 **(b),** S365 **(c),** and S373 **(d)** as well as total Cx43 on proteins extracted from left ventricular (LV) SSM of wildtype (WT) and Cx43^{PKCmut} mice. Both original Western Blot images (upper panels) and data in which the ratio of phosphorylated over total Cx43 is shown (lower panels, both individual data points and mean values ± SEM) are presented. *: $p<0.05$ (unpaired t-test).

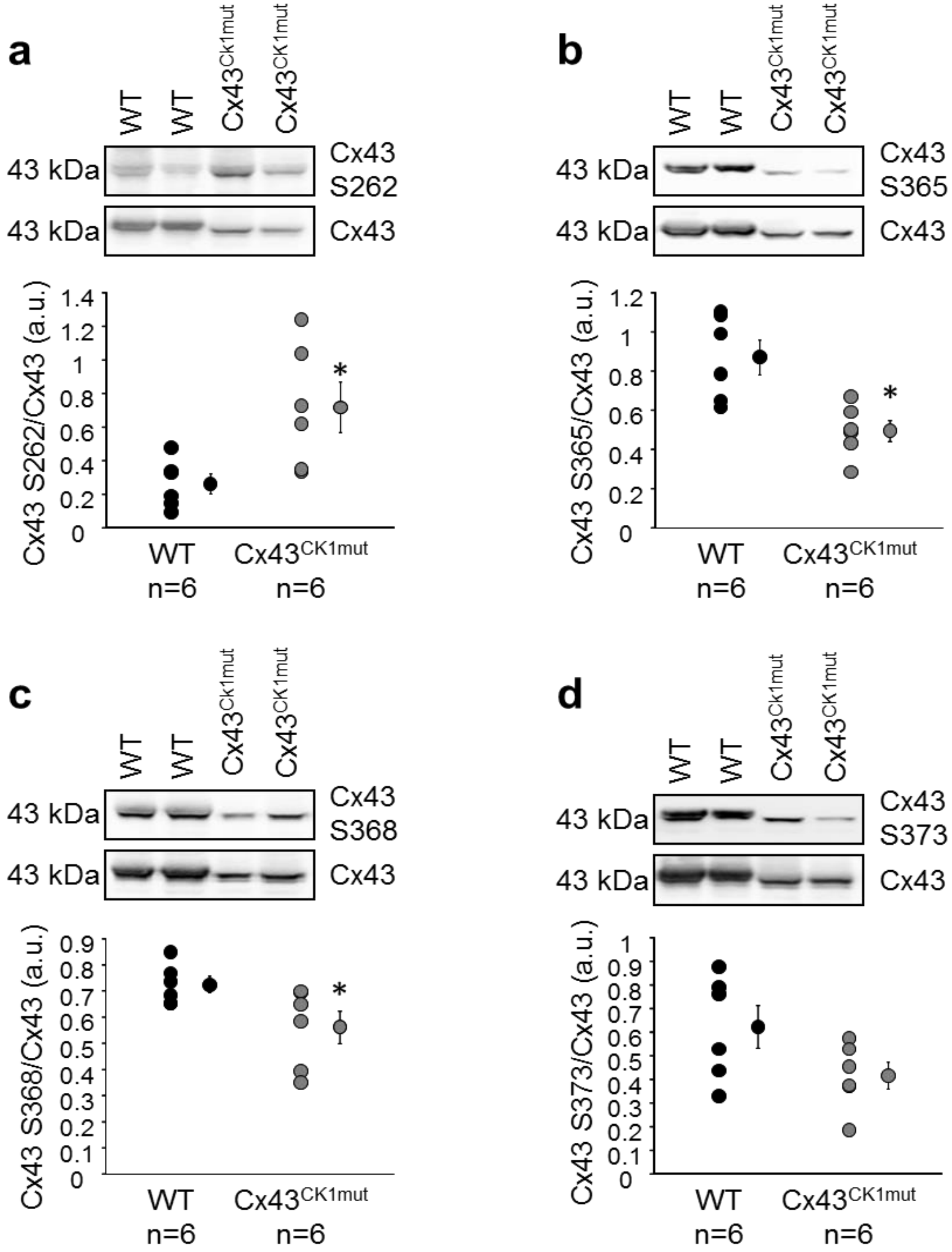

Supplementary figure 10 Phosphorylation of Cx43 in LV protein samples in WT and Cx43^{CK1mut} mice
Western Blot analysis was performed for Cx43 phosphorylated at S262 (**a**), S365 (**b**), S368 (**c**), and S373 (**d**) as well as total Cx43 on proteins extracted from left ventricular (LV) tissue of wildtype (WT) and Cx43^{CK1mut} mice. Both original Western Blot images (upper panels) and data in which the ratio of phosphorylated over total Cx43 is shown (lower panels, both individual data points and mean values ± SEM) are presented. *: p<0.05 (unpaired t-test).

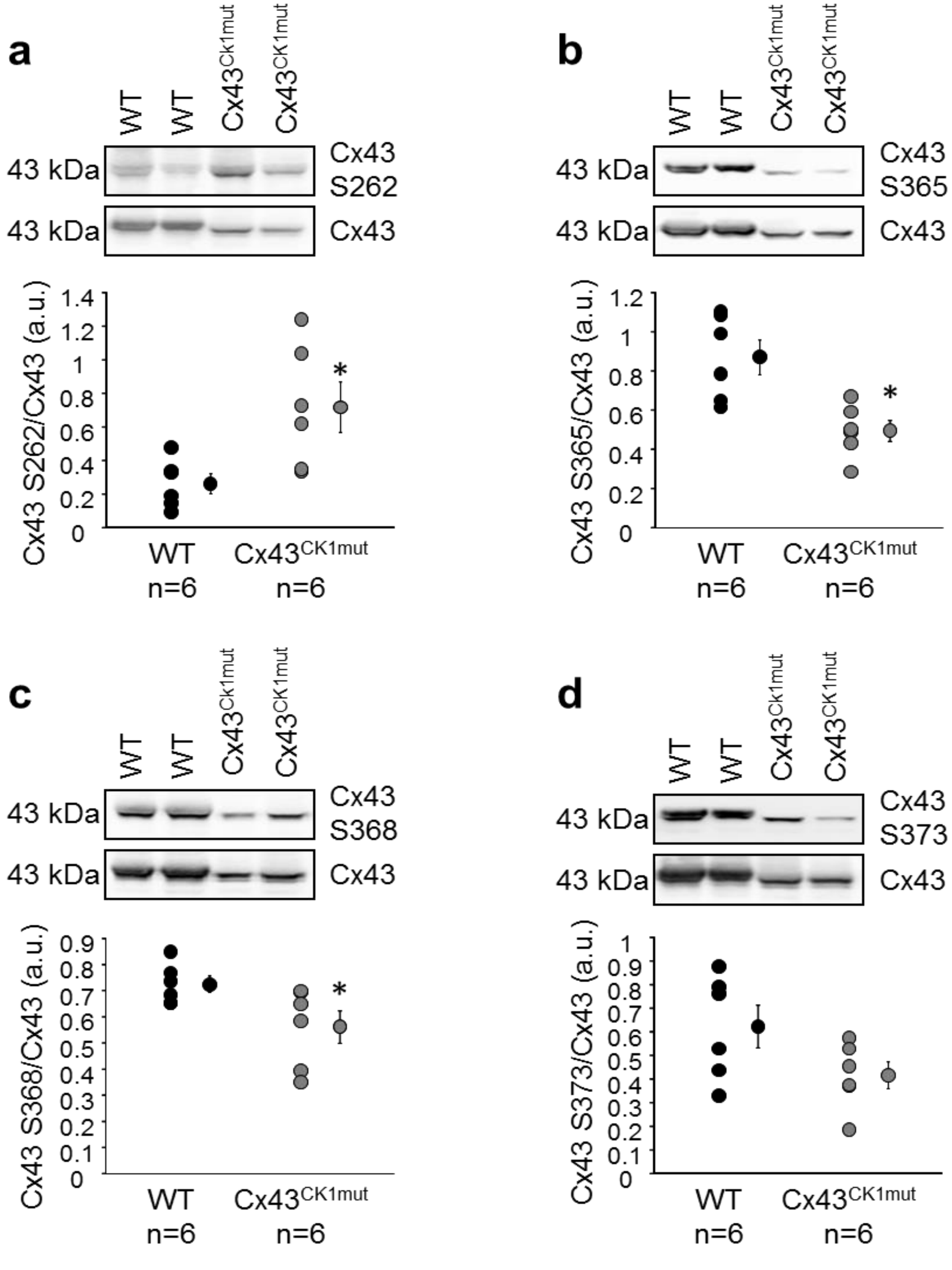

Supplementary figure 11 Cx43 phosphorylation in SSM in WT and $Cx43^{CK1mut}$ mice

Western Blot analysis was performed for Cx43 phosphorylated at S262 (**a**), S365 (**b**), S368 (**c**), and S373 (**d**) as well as total Cx43 on proteins extracted from left ventricular (LV) SSM of wildtype (WT) and $Cx43^{CK1mut}$ mice. Both original Western Blot images (upper panels) and data in which the ratio of phosphorylated over total Cx43 is shown (lower panels, both individual data points and mean values ± SEM) are presented,* $p<0.05$ (unpaired t-test).

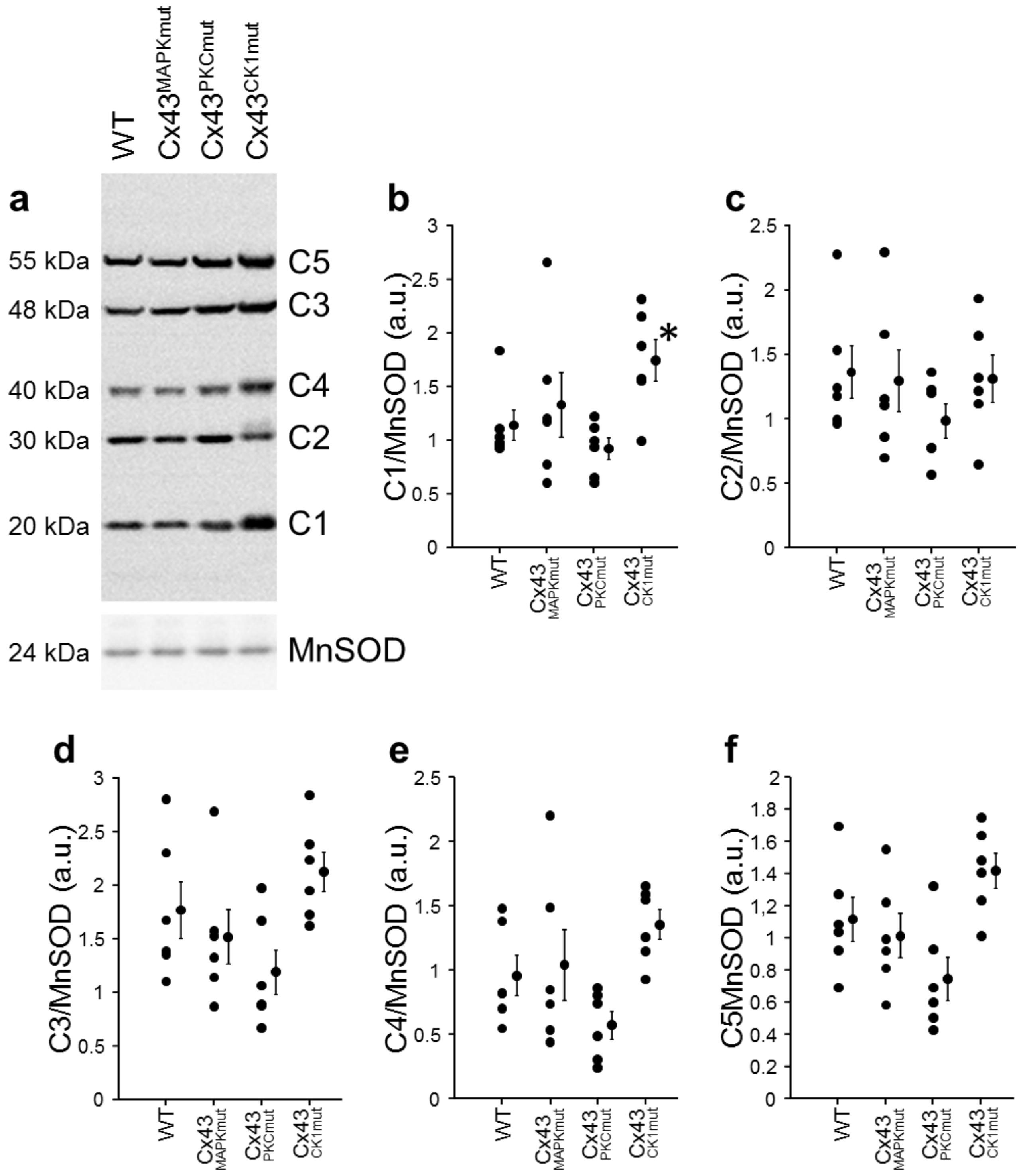

Supplementary figure 12 Expression of electron transport chain complexes in WT, Cx43^{MAPKmut}, Cx43^{PKCmut} and Cx43^{CK1mut} mice

Western blot analysis was performed for marker proteins of the electron transport chain complexes 1-5 (C1-C5) on left ventricular (LV) total protein extracts of wildtype (WT), Cx43^{MAPKmut}, Cx43^{PKCmut} and Cx43^{CK1mut}

mice. The mitochondrial marker protein manganese superoxide dismutase (MnSOD) was used for normalization (**a**). Diagrams indicate the protein levels of complex 1 (**b**), complex 2 (**c**), complex 3 (**d**), complex 4 (**e**) and complex 5 (**f**) normalized to MnSOD in arbitrary units (a.u., n=6 for each genotype). Both the single values for each mouse as well as the mean values ±SEM are presented. * $p<0.05$ vs. WT (unpaired t-test).

References

1. Cooper CD, Lampe PD (2002) Casein kinase 1 regulates connexin-43 gap junction assembly. J Biol Chem 277:44962-44968 doi:10.1074/jbc.M209427200
2. Hirschhäuser C, Sydykov A, Wolf A, Esfandiary A, Bornbaum J, Kutsche HS, Boengler K, Sommer N, Schreckenberg R, Schlüter KD, Weissmann N, Schermuly R, Schulz R (2020) Lack of contribution of p66shc to pressure overload-induced right heart hypertrophy. Int J Mol Sci 21: 9339 doi:10.3390/ijms21249339
3. Park JH, Lee MY, Heo JS, Han HJ (2008) A potential role of connexin 43 in epidermal growth factor-induced proliferation of mouse embryonic stem cells: involvement of Ca2+/PKC, p44/42 and p38 MAPKs pathways. Cell Prolif 41:786-802 doi:10.1111/j.1365-2184.2008.00552.x
